# Dye-Sensitized Solar Cells Based on the Principles and Materials of Photosynthesis: Mechanisms of Suppression and Enhancement of Photocurrent and Conversion Efficiency

**DOI:** 10.3390/ijms10114575

**Published:** 2009-10-27

**Authors:** Yasushi Koyama, Takeshi Miki, Xiao-Feng Wang, Hiroyoshi Nagae

**Affiliations:** 1 Faculty of Science and Technology, Kwansei Gakuin University, Sanda, Hyogo 669-1337, Japan; 2 Graduate School of Engineering, Gifu University, Yanagido, Gifu 501-1193, Japan; 3 Kobe City University of Foreign Studies, Gakuen Higashimachi, Nishi-ku, Kobe 651-2187, Japan

**Keywords:** dye-sensitized solar cells (DSSCs), photosynthesis, carotenoids, chlorophylls, pheophorbides, singlet-triplet annihilation, electron transfer, energy transfer, electron injection, charge recombination, co-sensitization

## Abstract

Attempts have been made to develop dye-sensitized solar cells based on the principles and materials of photosynthesis: We first tested photosynthetic pigments, carotenoids (Cars), chlorophylls (Chls) and their derivatives, to find sensitizers showing reasonable performance (photocurrent and conversion efficiency). We then tried to introduce the principles of photosynthesis, including electron transfer and energy transfer from Car to Phe *a*. Also, we tried co-sensitization using the pheophorbide (Phe) *a* and Chl *c*_2_ pair which further enhanced the performance of the component sensitizers as follows: *J*_sc_ = 9.0 + 13.8 → 14.0 mA cm^−2^ and *η* = 3.4 + 4.6 → 5.4%.

## 1. Introduction

Bacterial photosynthesis has been studied extensively: the structures of pigment-protein complexes were determined by X-ray crystallography and the excited-state dynamics of photosynthetic pigments, *i.e.*, carotenoids (Cars) and bacteriochlorophylls (BChls), by time-resolved laser spectroscopies in relation to their physiological functions. The goal of primary processes of photosynthesis is to generate the source of chemical energy, ATP, and the reductant, NADPH. However, the initial process of photosynthesis is to trigger the electron-transfer reaction by the use of harvested light energy. Therefore, the principles and the materials of photosynthesis can be used to fabricate dye-sensitized solar-cells (DSSCs).

In this mini-review, we will try to reorganize the results of our eight years of investigation, and to present our recent results, as well. At the beginning, we will introduce photosynthetic pigments and the principles of bacterial photosynthesis to those readers who have been studying DSSCs but not familiar with photosynthesis.

### 1.1. Photosynthetic Pigments

*Carotenoids*. The physiological functions of Cars include light-harvesting and photo-protection. The light-harvesting function of Cars includes the absorption of the light energy followed by singlet-energy transfer to BChl, which takes place in antennas including the peripheral LH2 and the central LH1 complexes. One of the photo-protective functions is the quenching of the lowest triplet (T_1_) BChl, which can sensitize the generation of harmful singlet oxygen. The other photo-protective function is the reduction of doublet ground-state radical-cation (D_0_^•+^) BChl to prevent its oxidative degradation.

**Figure 1.**
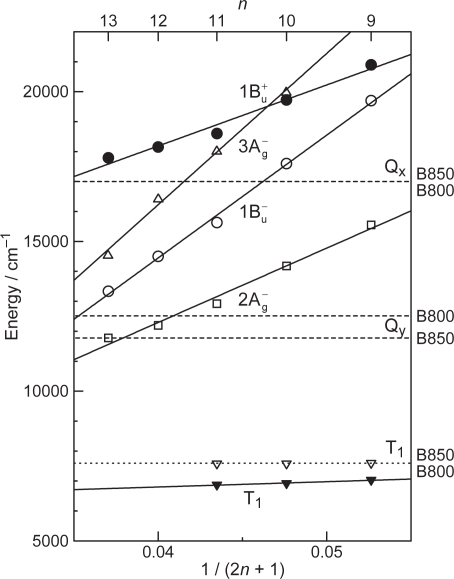
A diagram comparing the energies of the singlet- and triplet-excited states of Cars with those of BChl *a*. The energies of the optically-allowed 1B_u_^+^ state and the optically-forbidden 3A_g_^−^, 1B_u_^−^ and 2A_g_^−^ states were determined by measurement of resonance-Raman excitation profiles of crystalline mini-*β*-carotene, spheroidene, lycopene, anhydrorhodovibrin and spirilloxanthin (*n* = 9~13) [1]. The T_1_ levels of the Cars (*n* = 9~11) and BChl *a* bound to LH2 antenna complexes were determined by high-sensitively emission spectroscopy [2] ([9]–reproduced by permission of The Royal Society of Chemistry).

Figure 1 presents an energy diagram comparing the singlet-excited states of Cars (1B_u_^+^, 3A_g_^−^, 1B_u_^−^ and 2A_g_^−^) and those of BChl *a* (Q_x_ and Q_y_). The energies of Car excited states decrease with the number of conjugated double bonds, *n*, as functions of 1/(2 *n* + 1) [1]. There are two different kinds of BChls in LH2, absorbing at 800 and 850 nm (named ‘B800’ and ‘B850’), while LH1, only ‘B880’. The relative heights of singlet-energy levels show that the most efficient singlet-energy transfer from Car to BChl can take place in Cars (*n* = 9 and 10) through three different channels (1B_u_^+^ → Q_x_, 1B_u_^−^ → Q_x_ and 2A_g_^−^ → Q_y_).

Thus, Cars can be used in DSSCs to facilitate (i) singlet-energy transfer to BChl, (ii) triplet-energy transfer from T_1_ BChl, and (iii) electron transfer to D_0_^•+^ BChl. In addition, Cars themselves can eject electron when an electron acceptor is available [3].

*Bacteriochlorophylls and Chlorophylls*. The physiological functions of BChls include singlet-energy transfer and the ejection and transfer of electron. The singlet energy that has been transferred from Car to BChl in LH2 through multi-channels can be transferred further to LH1 and, then, to the reaction center (RC) using the Q_y_ excitation of BChl. When the singlet Q_y_ energy reaches ‘the special-pair’ BChl_2_ (P), an electron is ejected. The initiation of electron transfer by the use of the Q_y_ energy of P is the most important event in bacterial photosynthesis.

**Figure 2.**
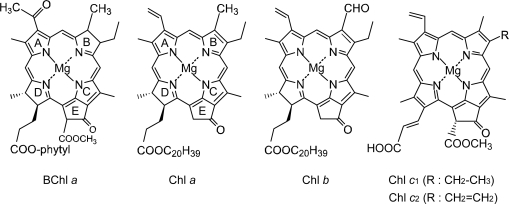
Chemical structures of BChl *a* having the bacteriochlorin skeleton, Chl *a* and Chl *b* having the chlorin skeleton, and Chl *c*_1_ and Chl *c*_2_ having the porphyrin skeleton.

The plants and algae use larger pigment-protein complexes, to which a large number of Car and chlorophyll (Chl) molecules are bound in more complicated ways. However, the basic principles of structural organization are similar to the pigment-protein complexes in photosynthetic bacteria. The uniqueness of these organisms is that they use different types of Chls including Chl *a*, Chl *b* and Chl *c*, the structures of which are shown in Figure 2 together with that of BChl *a*. The three different Chls can be characterized by their location and function as follows: (i) Chl *a* is the most common plant Chl, taking part in the light-harvesting pigment-protein complexes of higher plants, algae, prochlorophytes and cyanobacteria, and mainly functioning as the primary electron donors in the photosystem (PS) I and II RCs and also as the first electron accepter in PS I RC. (ii) Chl *b* is much less ubiquitous than Chl *a*. It is only present in the light-harvesting complexes, that are not closely connected to the RCs, of higher plants, green algae, euglenophytes and prochlorophytes. The main difference in its electronic-absorption spectrum from that of Chl *a* is the red shift of the Soret absorption and the blue shift of the Q_y_ absorption; the intensity of the latter relative to that of the former is much less in Chl *b* than in Chl *a*. (iii) Chl *c* was originally isolated from various marine algae as a mixture of closely-related pigments, Chl *c*_1_ and Chl *c*_2_. They have the porphyrin macrocycle (in contrast to Chl *a* and Chl *b* having the chlorin macrocycle) and have acrylic acid (instead of propionic acid ester) attached to ring D and the carboxyl methyl ester attached to ring E. Chl *c* exhibits a very strong Soret absorption shifted to the lower energy and a pair of very weak Q_y_ absorptions shifted to the higher energy (when compared to Chl *a*). Their main function is to mediate energy transfer from a carotenoid, fucoxanthin, to Chl *a* [4]. Thus, either Chl *b* or Chl *c* can transfer singlet energy to Chl *a* via the Q_y_ state, and function as supplementary light-harvesting pigments for Chl *a*, facilitating the initial electron-transfer reaction in the nonbacterial photosynthetic organisms.

### 1.2. How to Apply the Principles of Photosynthesis to DSSCs

**Figure 3.**
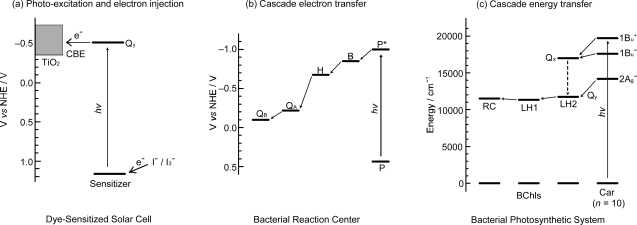
(a) Photo-excitation followed by electron injection and electron transfer in DSSC; (b) photo-excitation of special pair BChls (P) followed by cascade electron transfer in a sequence, special pair BChl_2_ (P) → accessory BChl (B) → bacteriopheophytin (H) → quinone A (Q_A_) → quinone B (Q_B_) in bacterial reaction center (RC); and (c) photo-excitation of Car to the optically-allowed 1B_u_^+^ state followed by energy transfer to the Q_x_ and Q_y_ levels of BChl, during the internal conversion processes of 1B_u_^+^ → 1B_u_^−^ → 2A_g_^−^ → G (1A_g_^−^) within Car in LH2 antenna. Then, the Q_y_ energy of BChl in the LH2 antenna is transferred to the LH1 antenna and eventually to P in the RC.

*Comparison between dye-sensitized solar cells and the bacterial photosynthetic system*. It is worthwhile to compare or contrast a typical Grätzel-type DSSC to the primary processes of bacterial photosynthesis (Figure 3), pictorially summarizing what have been described above: (a) Photo-excitation and electron injection in DSSCs: The assembly and principle of Grätzel-type DSSC is rather simple: A dye sensitizer is bound, through an anchoring group, to the surface of a semi-conductor, sintered TiO_2_ nanoparticles, for example, which can tremendously increase the area of the boundary surface. Upon photo-excitation of the sensitizer, electron is injected into TiO_2_ (to be transferred to the cathode) and the resultant dye radical cation is neutralized by the I^−/^I^−^_3_ redox couple (by transferring electron from the anode). The dye molecules that are piled up above the first layer can collect the light energy and transfer their singlet excitation (functioning as an antenna) and, at the same time, dissipate the singlet and triplet energies (functioning as a self-quencher). (b) Cascade electron transfer in the bacterial reaction center (RC): After charge separation at the special-pair BChls (P), triggered by photo-excitation, the electron is transferred to accessory BChl (B), bacteriopheophytin (H), quinone A (Q_A_) and eventually to quinone B (Q_B_). The locations, orientations, and the one-electron oxidation potentials of the series of electron-transfer components are finely tuned by intermolecular interaction with the apo-peptide(s) and other pigment(s). (c) Cascade energy transfer in the bacterial photosynthetic system: Cars harvest the light energy (in the 500 nm region) as supplementary light harvester and transfer their singlet energy to BChls through plural channels. Then, the singlet energy of BChl is transferred in the order, LH2 → LH1 → RC. In the Car → BChl energy transfer, both the optically-allowed 1B_u_^+^ and optically-forbidden 1B_u_^−^ and 2A_g_^−^ states of Cars as well as the optically-allowed Q_x_ and Q_y_ states of BChls are involved (see Figure 1), whereas in the latter BChl → BChl energy transfer, only through the lowest Q_y_ state of BChl, is involved.

*Strategies to be taken.* We know that Cars and Chls (including their derivatives) have the potential of electron injection into TiO_2_, upon photo-excitation, when they are bound directly to the linear or cyclic π-conjugated systems through the anchoring carboxyl group. We have started with using Cars as sensitizers, because we have accumulated knowledge concerning their excited-state energetics and dynamics (vide infra). Then, we proceeded to Chl and derivatives, in which the excited-state energy levels had been described by Gouterman [5,6] and excited-state dynamics had been studied by other investigators [7]. We first tried to learn the mechanisms how these photosynthetic pigments can function as the sensitizers in DSSCs, by systematically changing the degree of π-conjugation that determines the excited-state and the redox-state properties, which have turned out to be the key parameters in suppressing or enhancing the photocurrent and conversion efficiency of DSSC.

We also tried to introduce to the DSSC systems the first steps of the cascade electron transfer and energy transfer. We tried to incorporate sequential co-sensitization, the electron transfer and energy transfer from the Car moiety to the pheophorbide sensitizer and, also, parallel co-sensitization by the use of pheophorbide and chlorophyll sensitizers both having the anchoring carboxyl group.

In this review, we will try to let Figures illustrate the ideas and the experimental results by themselves, minimizing the lengths of sentences for explanation. We will briefly introduce the topics at the beginning and add a brief summary at the end, to facilitate the readers’ understanding. After Conclusion and Future Perspective, we will briefly introduce “Relevant Work by Other Investigators” to benefit the readers in evaluating our contribution.

## 2. Polyene Sensitizers

**Figure 4.**
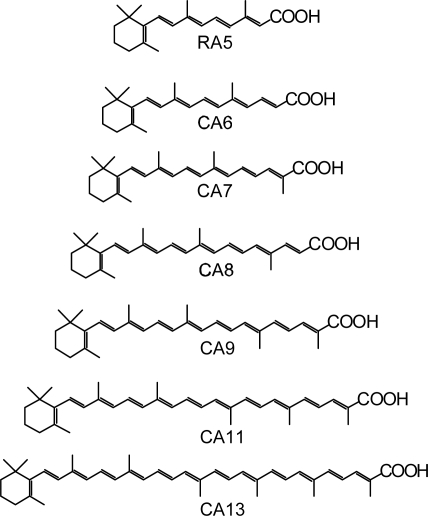
Chemical structures of retinoic acid (RA5) and carotenoic acids (CA6~CA13).

Polyenes are linear conjugated systems, from which electron can be injected into TiO_2_, when the carboxyl group is directly attached to facilitate binding and electron injection. As a set of sensitizers, we used retinoic acid (RA) and carotenoic acids (CAs) having *n* = 5~13 double bonds (Figure 4). Their dependence of excited-state energetics and dynamics on the conjugation length (*n*) has been well-documented [8,9]. Their one-electron oxidation potential shifts with *n* to the negative side (to the higher energy) systematically (*vide infra*).

We first examined the conjugation-length (*n*) dependence of the photocurrent and conversion efficiency (sometimes collectly called ‘performance’) of solar cells using the set of sensitizers, and tried to explain the results in terms of the excited-state dynamics of RA and CAs free in solution and bound to TiO_2_ nanoparticles in suspension. The maximum performance was obtained in CA7; the decline of performance toward CA13 was explained by the initial electron-injection efficiency, whereas the decline toward RA5 was partially explained in terms of triplet generation at later stages after excitation.

Secondly, we examined the concentration dependence of the performance of CA7-sensitized solar cell by dilution of the sensitizer with a spacer, deoxycholic acid (DCA). Surprisingly, the performance was enhanced from that at 100% by the initial dilution to 70% and even after the later dilution to 30%. The concentration dependence of the IPCE profile and the electronic absorption spectrum suggested changes in the form of singlet excitation of the sensitizer on the TiO_2_ layer. We suspected that ‘singlet-triplet annihilation’ due to the aggregate formation is the key in suppressing the photocurrent and conversion efficiency before dilution.

Finally, we prepared a set of four sensitizers having different polarizabilities and, as a result, different tendency of aggregated formation, and examined changes in the photocurrent and conversion efficiency of the fabricated solar cells, depending on the dye concentration and the light intensity. The most aggregate-forming dye exhibited the enhancement of performance by lowering the concentration and the light intensity, supporting the idea of singlet-triplet annihilation. The details will be described below.

### 2.1. Mechanisms of Electron Injection and Charge Recombination Generating Radical Cation and Triplet Species

**Figure 5.**
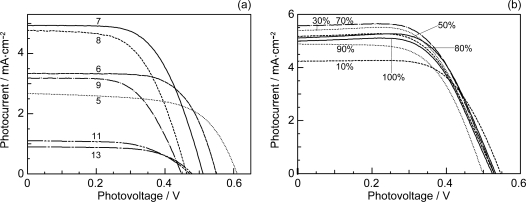
(a) Conjugation-length (*n*) dependence of the *I*–*V* curves in solar cells using the RA and CA sensitizers, and (b) the concentration dependence of the *I*–*V* curve in CA7-sensitized solar cells (reprinted from [10], Copyright (2005), with permission from Elsevier).

*Conjugation-length dependence of photocurrent and conversion efficiency of RA- and CA-sensitized solar cells.* Figure 5a shows the *I–V* curves of solar cells using the set of sensitizers [10]. The short-circuit photocurrent density (*J*_sc_) is in the order, RA5 < CA6 < CA7 > CA8 > CA9 > CA11 > CA13, whereas the open-circuit photovoltage (*V*_oc_) is in the order, RA5 > CA6 > CA7 > CA8, and CA8, CA9, CA11 and CA13 exhibit similar values.

**Figure 6.**
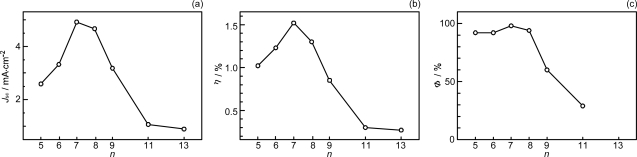
Conjugation-length (*n*) dependence of (a) the photocurrent (*J*_sc_) and (b) the conversion efficiency (*η*) in solar cells using the RA and CA sensitizers, and (c) the electron-injection efficiency (*Φ*) in the RA and CA sensitizers bound to TiO_2_ nanoparticles in suspension (reprinted with permission from [11] ^©^ 2005, American Chemical Society).

Presumably, the coverage on the surface of TiO_2_ layer should be better-organized in the shorter-chain RA5, CA6 and CA7 sensitizers in the complete all-*trans* configuration; the longer-chain sensitizers tend to form *cis* isomers, as well. Open-circuit photovoltage (*V*_oc_) in Figure 5 must reflect this situation. Figures 6a and b present the conjugation-length dependence of short-circuit current density (*J*_sc_, hereafter called ‘photocurrent’) and solar energy-to-electricity conversion efficiency (*η*, called ‘conversion efficiency’). Both photocurrent and conversion efficiency are at the maximum in CA7; they decline toward the shorter-chain in the order, CA6 and RA5, and also toward the longer-chain in the order, CA8, CA9, CA11 and CA13. The relevant parameters of solar cells and the one-electron oxidation potentials of the sensitizers are listed in Table 1 in Supporting Information of Ref. [10].

**Figure 7.**
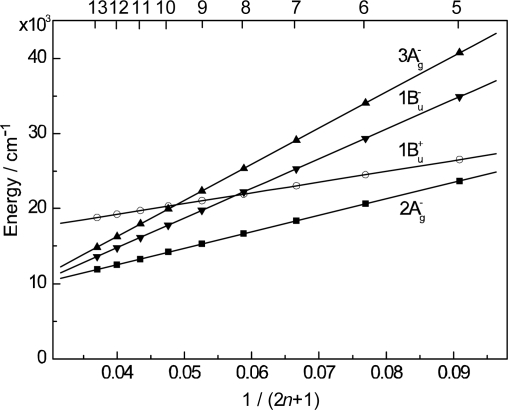
An energy diagram for the optically-allowed 1B_u_^+^ and optically-forbidden 2A_g_^−^, 1B_u_^−^ and 3A_g_^−^ states for conjugated chains having *n* = 5~13 double bonds (reprinted with permission from [11] ^©^ 2005, American Chemical Society).

*The excited-state dynamics of RA and CAs bound to TiO_2_* *nanoparticles in suspension.* To understand the mechanism giving rise to the above dependence of photocurrent and conversion efficiency on *n*, we examined the excited-state dynamics of the set of sensitizers (except for CA13) bound to TiO_2_ nanoparticles in suspension by subpicosecond and submicrosecond pump-probe spectroscopy [11]: Figure 7 shows an energy diagram for the π-conjugated chains of RA and CAs with *n* = 5~13: The linear dependence of the optically-active 1B_u_^+^ state, as a function of 1/(2*n* + 1), was determined by conventional electronic-absorption spectroscopy. The linear dependence of the optically-forbidden 1B_u_^−^, 3A_g_^−^ and 2A_g_^−^ states was transferred from those of bacterial Cars (*n* = 9~13) determined by the measurement of resonance-Raman excitation profiles [1] (Figure 1); the energies for CA8~RA5 were extrapolation of the linear relations. According to the state ordering, after excitation to the 1B_u_^+^ state by the absorption of photon, (i) RA5, CA6, CA7 and CA8 are expected to internally convert, in the order, 1B_u_^+^ → 2A_g_^−^ → 1A_g_^−^ (the ground state), (ii) CA9 and CA10, in the order, 1B_u_^+^ → 1B_u_^−^ → 2A_g_^−^ →1A_g_^−^ and (iii) CA11, in the order, 1B_u_^+^ →3A_g_^−^ → 1B_u_^−^ → 2A_g_^−^ → 1A_g_^−^.

On the basis of the above set of energy levels and internal conversion processes, we analyzed, by means of singular-value-decomposition (SVD) followed by global fitting, the time-resolved data matrices for the set of RA5~CA11 sensitizers free in solution and bound to TiO_2_ nanoparticles in suspension.

**Figure 8.**
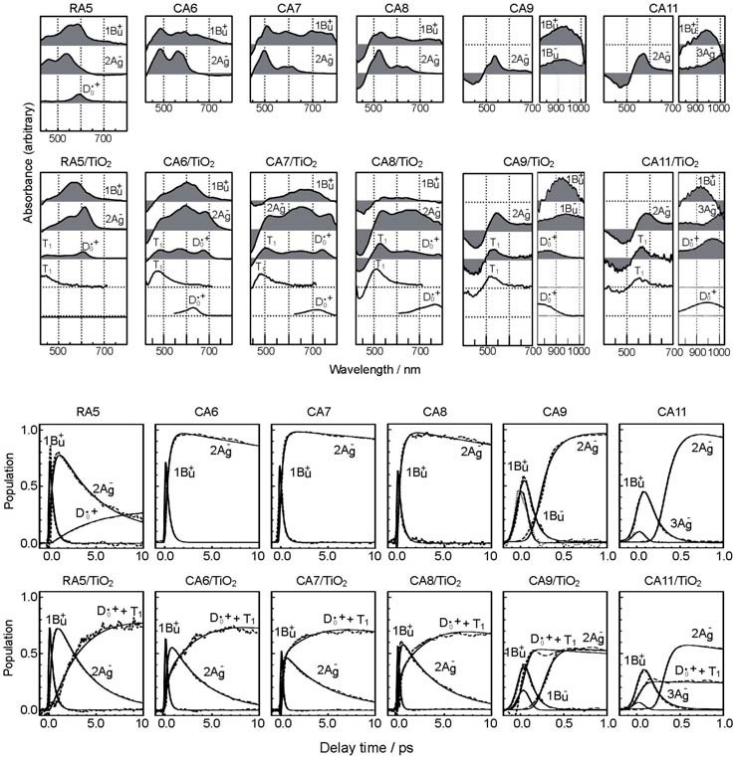
SADS and time-dependent changes in population for the RA and CA sensitizers free in ethanol solution (the first and the third panels) and bound to TiO_2_ nanoparticles in suspension (the second and the fourth panels) (reprinted with permission from [11] ^©^ 2005, American Chemical Society).

Figure 8 presents the results for free in solution, including the species-associated-difference-spectra (SADS) shown in the top panels and the time-dependent changes in population shown in the third panels: In RA 5, rapid transformation from the 1B_u_^+^ to the 2A_g_^−^ state followed by the generation of radical cation (D_0_^•+^) is observed. In CA6~CA8, rapid 1B_u_^+^ → 2A_g_^−^ transformation followed by the slow decay of the 2A_g_^−^ state is observed; here, no generation of D_0_^•+^ is seen. In the SADS of CA9 and CA11, clear transformation from the 1B_u_^+^ to the 2A_g_^−^ state is not seen in the visible region, but rapid transformation from the 1B_u_^+^ to the 1B_u_^−^ state and that from the 1B_u_^+^ to the 3A_g_^−^ state, respectively, are seen in the near-infrared region. Their spectral patterns agreed with those of the 1B_u_^−^ and 3A_g_^−^ states of neurosporene (*n* = 9) and lycopene (*n* = 11), respectively [12]. The time-dependent changes in population for CA9 shows extremely-rapid 1B_u_^+^ → 1B_u_^−^ transformation followed by the slower 1B_u_^−^ → 2A_g_^−^ transformation, whereas those for CA11, extremely-rapid 1B_u_^+^ → 3A_g_^−^ transformation followed by the slower 3A_g_^−^ → 2A_g_^−^ transformation.

The results for RA5~CA11 bound to TiO_2_ nanoparticles in suspension are also shown in Figure 8 (the second and fourth panels): The singlet-excited states generated by the photo-excitation of the sensitizers bound to TiO_2_ were basically the same as those generated free in solution. The most conspicuous difference in the excited-state dynamics, in the bound state, is that the transient absorptions of the triplet (T_1_) and the radical-cation (D_0_^•+^) states appear immediately after electron injection. The former transient absorptions agree in energy with those of the T_1_ states obtained by anthracene-sensitized photo-excitation, whereas the latter transient absorptions, with the stationary-state absorptions of radical cation obtained electrochemically (see the spectral lines shown in the second panels). The generation of the apparent D_0_^•+^ + T_1_ state, however, drastically influences the dynamics of singlet-excited states: In RA5~CA8, the generation of the D_0_^•+^ + T_1_ state substantially accelerates the decay of both the 1B_u_^+^ and 2A_g_^−^ states, showing efficient electron injection from these excited states into TiO_2_. In CA9 and CA11, on the other hand, it accelerates the decay of *not* the 2A_g_^−^ state *but* the 1B_u_^+^ state, showing electron injection only from the latter.

**Figure 9.**
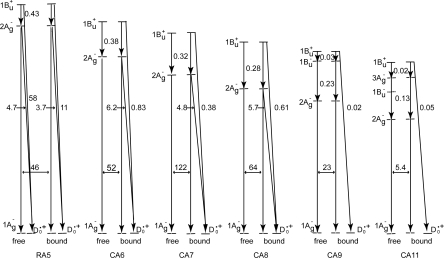
The pathways of internal conversion and electron injection for the RA and CA sensitizers free in solution and bound to TiO_2_ nanoparticles in suspension. The time constant for each pathway is shown in picoseconds (reprinted with permission from [11] ^©^ 2005, American Chemical Society).

Figure 9 presents the internal-conversion and electron-injection pathways and the relevant time constants for the free and bound states. Table 1 lists the electron-injection efficiencies through the 1B_u_^+^ and 2A_g_^−^ channels and a sum of the two for the set of RA and CAs, which were calculated by the use of those time constants.

The conjugation-length dependence of the total electron-injection efficiency (*Φ*) is depicted in Figure 6c. The highest efficiency in CA7 (almost unity) and the decline toward CA11 can be explained nicely in terms of electron-injection efficiency. The results definitely indicate that the decline toward the longer-chain, *i.e.*, CA7 > CA8 > CA9 > CA11, reflects the intrinsic excited-state dynamics of the Car conjugated chain. However, the decline toward CA6 and RA5 is left unexplained. Table 2 shows that the values of one electron-oxidation potential systematical lowers with *n*, a trend which predicts the electron-injection efficiency monotonically increasing with *n* all the way from *n* = 5 to 11, which is contrary to the observation.

We have observed the generation of ‘the D_0_^•+^ + T_1_ state’ just by transient absorptions, which does not decay at all in the ps time scale. Therefore, we do not know, at this moment, what we now call ‘the D_0_^•+^ + T_1_ state’ is either ‘a *combined* D_0_^•+^ + T_1_ state’ or ‘a *mixture* of the D_0_^•+^ state and the T_1_ state’. We have applied submicrosecond pump-probe spectroscopy to examine the later stages after excitation.

**Figure 10.**
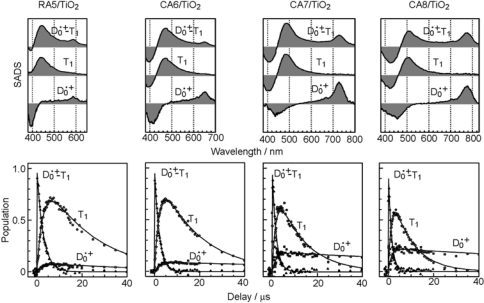
SADS (upper panels) and time-dependent changes in population (lower panels) for RA5~CA8 bound to TiO_2_ nanoparticles in suspension (reprinted with permission from [11] ^©^ 2005, American Chemical Society).

Figure 10 shows the results of the SVD and global-fitting analysis of submicrosecond time-resolved data matrices for the four shorter-chain RA and CAs. Here, a relaxation mechanism, including the splitting of a combined D_0_^•+^ + T_1_ state into a pair of the D_0_^•+^ and T_1_ states, has been nicely explained. The first SADS (upper panels) show that the T_1_/D_0_^•+^ population ratio in the *combined* D_0_^•+^ + T_1_ state increases toward RA5. Consistently, the time-dependent changes in population (lower panels) show that the ratio of the *split* T_1_/D_0_^•+^ species also increases toward RA5.

Table 3 lists the quantum yields for the D_0_^•+^ and T_1_ species (*ϕ*_D_ and *ϕ*_T_) calculated by the use of the relevant time constants. The efficiency of electron injection (*ϕ*_D_) gradually declines toward RA5. This trend *partially* solves the above-mentioned contradiction in the dependence on *n* shown in Figure 6, *i.e.*, (a) and (b) *vs* (c).

**Figure 11.**
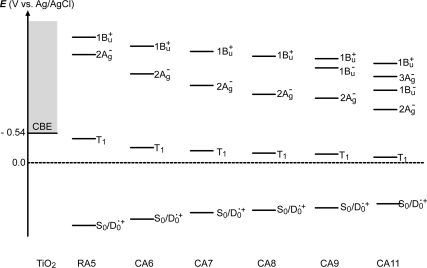
The energies of the singlet, triplet and redox states of RA5 and CA6~CA11 in reference to that of the conduction-bond edge (CBE) of TiO_2_. The redox (S_0_/D_0_^•+^) levels were drawn based on the one-electron oxidation potential listed in Table 2, and the excited state levels were taken from these shown in Scheme 7. Here, the T_1_ energy is assumed to be the 1/2 of the 2A_g_^−^ energy [13]. On the other hand, the energy of CBE was calculated by E_CBE_ = −0.1–0.059 pH (in eV) [14], where the pH value was assumed to be 3.0 (reprinted with permission from [11] ^©^ 2005, American Chemical Society).

Finally, we will propose the mechanisms of charge-separation and charge-recombination, which generates the radical-cation and triplet species of RA and CAs on the surface of TiO_2_ nanoparticles: Figure 11 presents the energies of the singlet, triplet and redox states of RA5 and CA6~CA11 in reference to the conduction-band edge (CBE) of TiO_2_. Importantly, the energy gap between the CBE and the T_1_ levels is the smallest in RA5 and systematically increases toward CA11, which explains the decreasing order of the triplet generation mentioned above.

**Figure 12.**
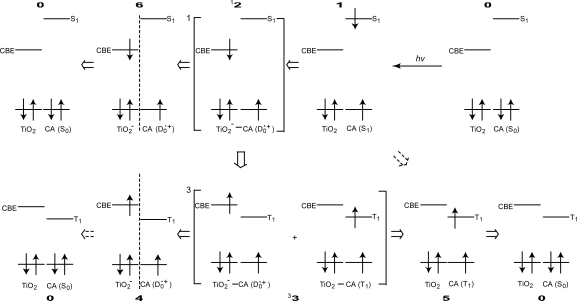
Excitation, electron transfer and relaxation dynamics in a typical Car bound to TiO_2_ nanoparticles in suspension. Mechanisms of electron injection as well as charge recombination, following intersystem crossing and exciplex formation, to generate triplet (T_1_) and radical cation (D_0_^•+^) species of the Car sensitizer. Each numbered state is expressed by a combination of TiO_2_ and CA in the ground, redox or excited states (reprinted with permission from [11] ^©^ 2005, American Chemical Society).

Figure 12 proposes the excited-state dynamics in a typical CA that is bound to TiO_2_: (i) Process **0** → **1**: Upon absorption of photon, electron is transferred to a higher singlet level (S_1_). (ii) Process **1** → ^1^**2**: Electron injection takes place to generate a charge-separated state having a singlet character on the boundary. (iii) ^1^**2** → **6**: Electron is transferred further into TiO_2_ to form a stable charge-separated state. (iv) **6** → **0**: the reverse electron transfer followed by charge recombination takes place to relax into the ground state. This is a series of changes among the singlet-excited and redox states having a singlet character.

Now, we will consider the generation of the triplet-excited and radicalcation states both having a triplet character: (v) Process ^1^**2** → ^3^**3**: When there is a strong spin-orbit coupling in the charge-separated state having the singlet character, it can transform, by the inversion of spin, into the charge-separated state having a triplet character. When the energy gap between the CBE and the T_1_ levels is small, the resultant charge-separated state can transform further into *a charge-transfer complex* (^3^**3**) consisting of the charge separated (TiO_2_^−^–CA(D_0_^•+^)) state and a neutral (TiO_2_–CA(T_1_)) state. This is exactly what we called ‘the *combined* D_0_^•+^ + T_1_ state’ (*vide supra*), because the former component gives rise to the radical-cation electronic absorption, whereas the latter component, the T_1_-state electronic absorption of CA.

In ^3^**3**, the relative contribution of the T_1_-state CA becomes larger when the energy gap between the CBE of TiO_2_ and the T_1_ states of CA becomes smaller (see Figure 11); this is actually evidenced by the SADS of the D_0_^•+^ + T_1_ state (see Figure 10). This charge-transfer complex can split into two independent components as follows: (vi) ^3^**3** → **4**: It transforms into the pure D_0_^•+^ state of CA, the lifetime of which can be very long when the electron is trapped far from the surface of TiO_2_ particles in suspension. (vii) ^3^**3** → **5**: it can transform into the T_1_ state of CA, which decays with an intrinsic T_1_ lifetime.

*Summary:* The mechanisms of electron injection immediately after excitation to the 1B_u_^+^(S_1_) state and charge recombination of the TiO_2_^−^–Car (D_0_^•+^) pair to form triplet Car, after the intersystem crossing and the formation of charge-transfer complex, have been revealed by the analysis of the ps and *μ*s time-resolved data obtained by pump-probe spectroscopy of RA and CAs bound to TiO_2_ nanoparticles in suspension. The conjugation-length (*n*) dependence of the initial excited-state dynamics has nicely explained the photocurrent and conversion efficiency of solar cells using the RA and CA sensitizers, *i.e.*, the maximum at *n* = 7 and the decline toward *n* = 11. On the other hand, the decline toward *n* = 5 has been explained *partially* in terms of the triplet generation at later stages.

### 2.2. Mechanisms of Singlet-Triplet Annihilation Suppressing Photocurrent and Conversion Efficiency

**Figure 13.**
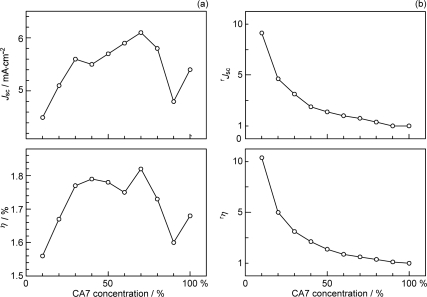
Effects of dilution of the CA7 sensitizer with a spacer, deoxycholic acid (DCA, the structure will be shown in Figure 18), on (a) the photocurrent (*J*_sc_) and conversion efficiency (*η*) and (b) the relative photocurrent (^r^*J*_sc_) and conversion efficiency (^r^*η*) of CA-sensitized solar cells. To obtain ^r^*J*_sc_(X) at a mole fraction X, for example, *J*_sc_(X) was scaled against concentration, and, then, a ratio was taken in reference to the value with no dilution. Thus, ^r^*J*_sc_(X) = *J*_sc_(X)/X/*J*_sc_(X = 1). By the same token, ^r^*η*(X) = *η*;(X)/X/*η*(X = 1) (reprinted from [10], Copyright (2005), with permission from Elsevier).

*Dependence of photocurrent and conversion efficiency on the dye concentration in CA7-sensitized solar cells: a possible mechanism of singlet-triplet annihilation.* Figure 5b (shown at the beginning of Section 2.1) presents the *I*–*V* curves of CA7-sensitized solar cells, when the sensitizer was diluted with a spacer, deoxycholic acid (DCA) [10]. Table 2 in Supporting Information of Ref. [10] lists the relevant parameters showing the performance of CA7-sensitized solar cells at different dye concentrations. Figure 13a shows the concentration dependence of *J_sc_* and *η*. Both parameters exhibit consistent but unique concentration dependence, which can be characterized as follows: (i) At 100%, these values are medium among the values at all the different concentrations. (ii) On going from 100% to 90%, the values exhibit a sudden drop. (iii) Then, they increase up to a maximum at 70%. (iv) From 70% down to 30%, the values gradually decrease. (v) Below 30%, they decrease steeply toward the values at 10%.

**Figure 14.**
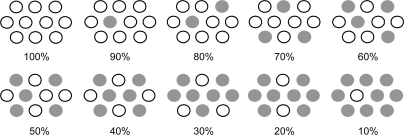
Typical arrangements of the dye (○) and spacer (•) molecules on the TiO_2_ surface formed during the processes of dilution of the former with the latter (reprinted from [10], Copyright (2005), with permission from Elsevier).

The consistent changes *not only* in photocurrent and conversion efficiency shown in Figure 13 *but also* in the IPCE profile (action spectrum) and the electronic absorption spectrum (see Ref. [10]) strongly suggest changes in the form of singlet excitation with the turning points at 90%, 70% and 30%. We propose four different forms of excitation based on Figure 14, where the dye molecules (○) are diluted with the spacer molecules (•): (i) At 100%, a coherent excitonic excitation takes place in an aggregate of dye molecules (we call this ‘coherent delocalized excitation’). (ii) At 90%, this excitation is destroyed by a small number of spacer molecules that function as defects. (iii) At 70%, a localized excitation on a single molecule can migrate from one to another. This ‘migrating excitation’ must become most efficient when the dye concentration becomes around 2/3, because branched routes for the migrating excitation are formed. (iv) At 30%, the dye molecules become isolated being intervened by a larger number of spacer molecules. This ‘isolated excitation’ must become the largest in number when the dye concentration becomes around 1/3.

Based on the above three different types of singlet excitation on the TiO_2_ layer and the generation of the triplet state as an intrinsic property of CAs bound to TiO_2_ (see Section 2.1), we propose a possible mechanism to explain the unique concentration dependence of photocurrent and conversion efficiency in the fabricated CA7-sensitized solar cell (see Figure 13a): (i) In a coherent delocalized excitation at 100%, there is a good chance that such widely-expanded excitation reaches a dye molecule in the T_1_ state to cause the singlet-triplet annihilation. (ii) In partially-destroyed delocalized excitation at 90%, the advantage of the widely-expanded coherent excitation in electron injection is lost to suppress electron injection, but there is still a chance of collision between ‘an expanded delocalized excitation’ and a localized triplet excitation to annihilate the former. (iii) In a localized excitation migrating along one of the branched routes at 70%, there is a much less chance of collision with a triplet excitation unless it is located on the particular route. (iv) In an isolated singlet excitation, there is no chance of collision with an isolated triplet excitation. Then, the photocurrent and conversion efficiency decrease linearly with the decreasing number of dye molecules excited.

The relative photocurrent (^r^*J*_sc_) and conversion efficiency (^r^*η*) are depicted in Figure 13b (see the caption for their definition). Their concentration dependence indicates that the changes in the singlet excitation take place continuously, and the relative performance (^r^*J*_sc_ and ^r^*η*) becomes systematically enhanced until 9~10 times on going from the first to the last form of singlet excitation.

*Summary*: The dependence of the photocurrent and conversion efficiency of the CA7-sensitizerd solar cell on the dye concentration has been explained in terms of changes in the form of singlet excitation of the sensitizer molecules on the surface of TiO_2_ layer, *i.e.*, the coherent delocalized excitation → the localized migrating excitation → the isolated excitation. There is a good chance of substantial enhancement of performance, if we succeeded in achieving only the localized excitation, keeping the total number of excited-state dye molecules the same.

The substantially reduced performance at the 100% dye concentration is ascribable to the singlet-triplet annihilation reaction. Therefore, the decrease in the photocurrent and conversion efficiency of solar cells from the CA7 sensitizer toward the RA5 sensitizer (see Figure 6a and b) can now be explained by the effect of singlet-triplet annihilation among the sensitizer molecules on the surface of the TiO_2_ layer, in addition to the effect of the increasing triplet generation described in Section 2.1.

*Dependence of conversion efficiency on dye concentration and light intensity in solar cells using polyene sensitizers having different polarizabilities*. Scheme 15 shows the structures of four different polyene sensitizers that were used for fabricating the solar cells [15]. The common skeleton of the sensitizers is the benzene ring connected to a polyene (*n* = 6), to the end of which the carboxyl group is attached (*ϕ*-6-CA); to the opposite end of the benzene ring the MeO-, (MeO)_3_- or Me_2_N- electron-donating groups is attached to realize the electron push-pull relation in the latter set of sensitizers.

The set of polyene sensitizers are named *ϕ*-6-CA, MeO-*ϕ*-6-CA, (MeO)_3_-*ϕ*-6-CA and Me_2_N-*ϕ*-6-CA as shown in the figure; the polarizability of polyene to enhance van der Waals intermolecular interaction to form aggregates is supposed to increase in this order. Actually, the transition-dipole moment calculated by the use of molar extinction coefficient (*ɛ*) was in the order, 14.2, 15.1, 15.2 and 15.6 D, and the tendency of aggregate formation judged by the blue-shift of the 1B_u_^+^ absorption was in the same order (data not shown).

**Figure 16.**
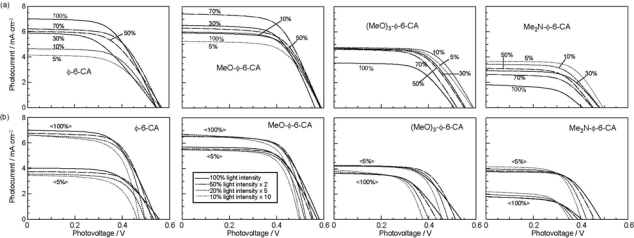
(a) The concentration dependence and (b) the light-intensity dependence (at two different concentrations) of the *I*–*V* curves in solar cells using the four sensitizers having different polarizabilities (reprinted from [15], Copyright (2006), with permission from Elsevier).

Figure 16a shows the concentration dependence of the *I*–*V* curves of solar cells using the above set of sensitizers. In the least-polarizable sensitizer, *ϕ*-6-CA, the photocurrent (*J*_sc_) is the highest at 100% and monotonously decreases toward the lower concentration. In the most-polarizable sensitizer, Me_2_N-*ϕ*-6-CA, on the other hand, the photocurrent is the lowest at 100% and monotonously increases toward the lower concentration. The latter change is contrary to our expectation, and can be explained only in terms of singlet-triplet annihilation. At 100%, the delocalized excitonic excitation should be generated due to the aggregate formation, which can be readily annihilated by collision with the triplet species within the expanded, excitonically-excited region. The chance of this singlet-triplet annihilation must become smaller by lowering the dye concentration.

Figure 16b shows the dependence of the *I*–*V* curves of the solar cells on the light intensity at two different dye concentrations (5% and 100%). In the least-polarizable sensitizer, *ϕ*-6-CA, the photocurrent decreases with the lowering light intensity. On the other hand, in the most-polarizable sensitizer, Me_2_N-*ϕ*-6-CA, the photocurrent increases, instead. The latter change is contrary to our expectation, and can be explained in terms of singlet-triplet annihilation, because the generation of both the singlet and triplet excitation must become suppressed at the lower light intensity.

**Figure 17.**
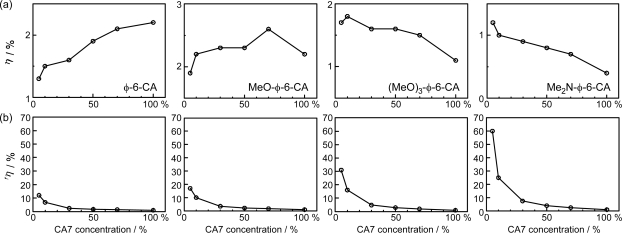
Concentration dependence of (a) the conversion efficiency (*η*), and (b) the relative conversion efficiency (^r^*η*) in solar cells using the four sensitizers with increasing polarizabilities. See the caption of Figure 13 for the definition of ^r^*η* (reprinted from [15], Copyright (2006), with permission from Elsevier).

Figure 17a plots the concentration dependence of conversion efficiency (*η*) for the set of polyene sensitizers. In the least-polarizable sensitizer, *ϕ*-6-CA, the conversion efficiency monotonously decreases, while in the most-polarizable sensitizer, Me_2_N-*ϕ*-6-CA, it monotonously increases with the lowering dye concentration. In the second-least polarizable sensitizer, MeO-*ϕ*-6-CA, conversion efficiency exhibits the maximum at 70%, while in the second-most polarizable sensitizer, (MeO)_3_-*ϕ*-6-CA, it exhibits the maximum at 5%.

Table 2 in Supporting Information of Ref. [15] lists the values of (i) conversion efficiency (*η*), (ii) conversion efficiency scaled to the concentration (^s^*η*), and (iii) the ratio of scaled conversion efficiency in reference to that at 100% (^r^*η*). The concentration dependence of the ^r^*η* values are depicted in Figure 8b. Interestingly, the relative conversion efficiency (^r^*η*) at 5% is in the order, Me_2_N-*ϕ*-6-CA > (MeO)_3_-*ϕ*-6-CA > MeO-*ϕ*-6-CA > *ϕ*-6-CA, in agreement with the decreasing order of polarizability of the sensitizers.

*Summary:* The absence or presence of singlet-triplet annihilation has been demonstrated by lowering the dye concentration and the light intensity in solar cells by the use of the four sensitizers having the increasing polarizability and, as a result, the increasing tendency of aggregate formation. The least polarizable (the least aggregate-forming) sensitizer gave rise to the decreasing conversion efficiency with the decreasing dye concentration and light intensity, whereas the most polarizable (the most aggregate-forming) sensitizer gave rise to the increasing conversion efficiency with the decreasing dye concentration and light intensity. The four different patterns, in the dependence on the dye concentration and the light intensity, can be used as a standard to examine the degree of aggregate formation and the absence and presence of singlet-triplet annihilation of a new sensitizer.

## 3. Pheophorbide Sensitizers Combined with Polyene Spacers

While searching for a sensitizer of Chl *a* derivative having a cyclic conjugated system, we found that pheophorbide *a* (Phe *a*) having the chlorin skeleton gave rise to reasonably-high photocurrent and conversion efficiency. As described in the previous section, a spacer is useful in preventing singlet-triplet annihilation due to aggregate formation of dye sensitizers, and, also, polyenes have high potential of electron injection, we have tried to use Phe *a* as the sensitizer and bacterial and plant Cars as redox spacers. Electron transfer from neutral Car to Phe *a* radical cation (Phe *a*^•+^) must prevent the charge recombination and stabilize the TiO_2_^−_^Car^•+^ charge-separated state. Actually, the Car spacers enhanced the photocurrent and conversion efficiency, and the above figure has been confirmed by subpicosecond pump-probe spectroscopy of Phe *a* and each bacterial Car bound to TiO_2_ nanoparticles in suspension.

We found no signs of singlet-energy transfer in the above experiments even by the use of the shortest-chain Cars having the higher singlet energies than those of Phe *a*. We suspected that the direct van der Waals contact and the correct orientation of transition dipoles between the Car and the Phe *a* moieties may be necessary to facilitate efficient singlet-energy transfer. We then synthesized an adduct sensitizer consisting of Phe *y* (modified from Phe *a*) and Car, which *actually* realized the singlet-energy transfer from the Car to the Phe moiety in addition to electron transfer, enhancing photocurrent and conversion efficiency. Further, the Car moiety, connected by single bonds to Phe *y*, prevented the aggregate formation and the resultant singlet-triplet annihilation, which was evidenced by the suppression of performance by lowering the light intensity. The details will be described below.

### 3.1. Mechanisms of Electron Transfer from Carotenoid Spacers to Pheophorbide a Sensitizer

**Figure 18.**
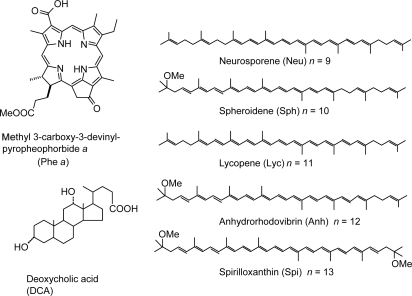
Chemical structures of the Phe *a* sensitizer and the spacers including DCA and a set of bacterial Cars with *n* = 9~13 (reprinted from [16], Copyright (2005), with permission from Elsevier).

*Phe a-sensitized solar cells using bacterial Cars as redox spacers.* Figure 18 presents the sensitizer, methyl 3-carboxyl-3-devinyl-pyropheophorbide *a* (hereafter, abbreviated as ‘Phe *a*’), and spacers, deoxycholic acid (DCA) and bacterial Cars including neurosporene, spheroidene, lycopene, anhydrorhodovibrin and spirilloxanthin (note the three-letter abbreviations) having *n* = 9, 10, 11, 12 and 13 conjugated double bonds. The sensitizer consists of chlorin conjugated macrocycle, to which the carboxyl group is directly attached to facilitate the binding and electron injection to TiO_2_ nanoparticles. DCA is a frequently-used saturated spacer with a carboxyl group, while Cars have no anchoring groups. Here, a 10% each of spacer was added to the sensitizer solution, in which the TiO_2_-deposited optically transparent electrode (OTE) was soaked overnight [16].

**Figure 19.**
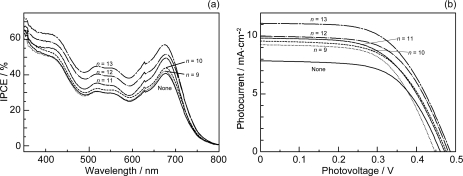
(a) The IPCE profiles and (b) the *I*–*V* curves of the Phe *a*-sensitized solar cells in the absence and presence of the Car redox spacer including neurosporene, spheroidene, lycopene, anhydrorhodovibrin or spirilloxanthin with *n* = 9~13 (reprinted from [16], Copyright (2005), with permission from Elsevier).

Figure 19 presents (a) the incident photon-to-current conversion efficiency (IPCE) profiles and (b) the *I*–*V* curves of Phe *a*-sensitized solar cells using Car redox spacers having different chain lengths (*n*); the solar cell with no spacers was also examined for comparison. Importantly, the patterns of IPCE profiles with and without Car spacers are basically the same, and no contribution of Car absorption is seen at all. Therefore, there is little chance of Car to Phe *a* singlet-energy transfer. The IPCE profile and the photocurrent in the *I*–*V* curve increase monotonously with the conjugation length (*n*) of the Car spacer.

Table 2 in Supporting Information of Ref. [16] lists the relevant parameters; the values of IPCE_670_ (the height at 670 nm), the integrated IPCE (∫*IPCE* d*v̄*), *J_sc_* and *η* increase with *n*. Accordingly, the one-electron oxidation potential (*E*_ox_) of Car shifts to the negative side with *n* (Table 1 of Ref. [16]).

**Figure 20.**
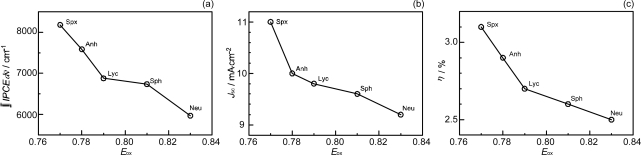
Dependence of the integrated IPCE (∫*IPCE* d*v̄*), photocurrent (*J*_sc_) and conversion efficiency (*η*) on the one-electron oxidation potential (*E*_ox_) of the Car spacer in the Phe *a*-sensitized solar cells.

Figure 20 depicts the correlations, ∫*IPCE* d*v̄*, *J*_sc_ and *η vs. E*_ox_. The correlations support our original idea of the neutralization of Phe *a*^•+^ with Car; spirilloxanthin (*n* = 13) with the lowest *E*_ox_ exhibits the highest potential of electron transfer.

To obtain spectroscopic evidence for the Car to Phe *a*^•+^ electron transfer, we performed subpicosecond pump-probe spectroscopy of the Phe *a* sensitizer and each Car spacer both bound to TiO_2_ nanoparticles in suspension [17]. The time constants of the Phe *a*^•+^ generation, as the result of electron injection to TiO_2_, are listed in Table 4; they have been determined by the SVD and global-fitting analysis of the data matrices in the 0.00–0.50 ps time region.

**Figure 21.**
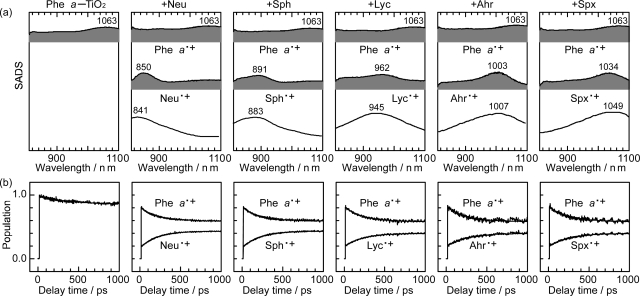
(a) SADS and (b) time-dependent changes in population, obtained by the SVD and global fitting analysis of the time-resolved data matrices in the 15 ps–1 ns region, for Phe *a* bound to TiO_2_ nanoparticles in suspension in the absence and presence of 10% each of neurosporene, spheroidene, lycopene, anhydrorhodovibrin and spirilloxanthin. The absorption peak of each Car^•+^ free in solution electrochemically generated, is presented as a line spectrum for comparison (reprinted from [17], Copyright (2005), with permission from Elsevier).

Figure 21a shows the results of SVD and global-fitting in the 15 ps–1 ns region. The transient absorption of each Car^•+^ obtained as SADS nicely agrees with its stationary-state absorption obtained by opto-electrochemistry (the line spectra). Thus, the assignment of Neu^•+^, Sph^•+^, Lyc^•+^, Ahr^•+^ and Spx^•+^ has been established. Each pair of time-dependent changes in population (Figure 21b) evidences electron transfer from the neutral Car to Phe *a*^•+^ to generate Car radical cation (Car^•+^). The time constants of electron transfer from each Car to Phe *a*^•+^ are listed in Table 4 as ‘Phe *a*^•+^ decay’; they are in the 200–240 ps region.

**Figure 22.**
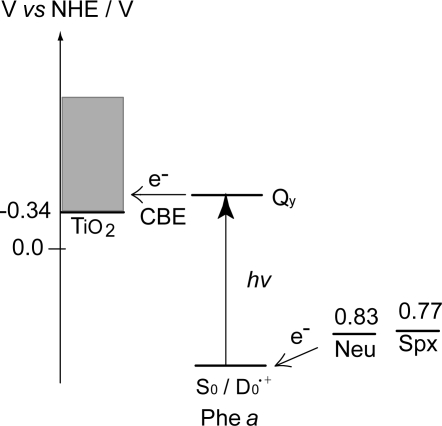
A mechanism of electron injection from Phe *a* to TiO_2_ followed by electron transfer from neurosporene (*n* = 9) or spirilloxanthin (*n* = 13). The gap in one-electron oxidation potential (in energy) between the relevant Car and Phe *a* determines the rate and the amount of electron transfer (reprinted from [17], Copyright (2005), with permission from Elsevier).

Figure 22 shows a mechanism of electron transfer: Spirilloxanthin having the lowest one-electron potential (the highest energy) most effectively promotes the electron transfer from Car to Phe *a*^•+^ and suppresses the reverse electron transfer in comparison to neurosporene having the highest one-electron potential (the lowest energy). On the other hand, the rate of resonance electron transfer is the highest in neurosporene, where energy gap to the S_0_/D_0_^•+^ level is the smallest.

**Figure 23.**
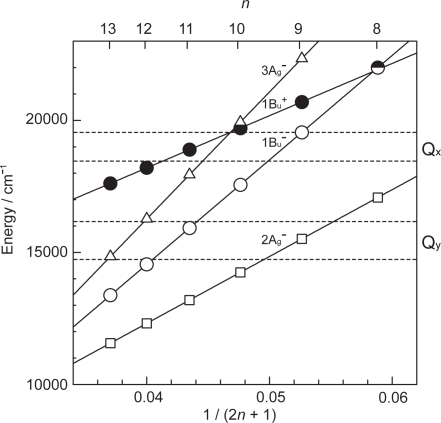
The energies of the optically-allowed 1B_u_^+^ and the optically-forbidden 2A_g_^−^, 1B_u_^−^ and 3A_g_^−^ states of Car and those of the Q_x_ and Q_y_ states of Phe *a* (Phe *y*). Shorter-chain Cars (*n* = 8 and 9) have a better chance of singlet-energy transfer to Phe *a*.

Now, we consider the reason why no Car-to-Phe *a* singlet-energy transfer took place: Figure 23 presents an energy diagram comparing the 1B_u_^+^, 1B_u_^−^ and 2A_g_^−^ singlet-excited states of bacterial Cars (*n* = 9~13) to the Q_x_ and Q_y_ states of Phe *a*. For Phe *a*, the Q_x_ and Q_y_ energies are too high to facilitate efficient singlet transfer for the longer-chain Cars (*n* = 10~13). The only exception is for the 1B_u_^+^ state of Car (*n* = 9), neurosporene. However, no indication of singlet-energy transfer has been found even in the IPCE profile of this particular Car. Then, we have decided to try shorter-chain plant Cars.

**Figure 24.**
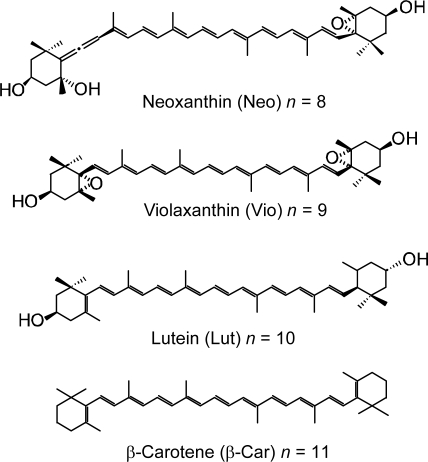
Chemical structures of plant Cars with *n* = 8~11.

*Phe a-sensitized solar cells using plant Cars as redox spacers.* Figure 24 presents the structures of plant Cars used as redox spacers, including neoxanthin, violaxanthin, lutein and *β*-carotene (note the three-latter abbreviations) with *n* = 8, 9, 10 and 11, respectively [18].

**Figure 25.**
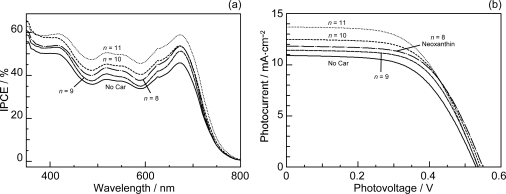
(a) The *IPCE* profiles and (b) the *I*–*V* curves in the Phe *a*-sensitized solar cells with no Cars and with a 20% each of Cars, including neoxanthin, violaxanthin, lutein and *β*-carotene (*n* = 8~11) (reprinted from [18], Copyright (2006), with permission from Elsevier).

The former three have polar pheripheral groups, while the last one is a symmetric hydrocarbon. Figure 25 shows (a) the IPCE profiles and (b) the *I*–*V* curves of solar cells using the Phe *a* sensitizer and the set of Car spacers. Importantly, the IPCE profile and the photocurrent (*J*_sc_) systematically shift to the higher values in the order, *n* = 9 < *n* = 8 < *n* = 10 < *n* = 11. Again, these is no clear indication of Car to Phe *a* energy transfer even in the shortest-chain Cars (*n* = 8 and 9). Table 3 of Ref, [18] lists relevant parameters concerning the performance of the solar cells. The *E*_ox_ values are listed in Table 1 of Ref. [18].

**Figure 26.**
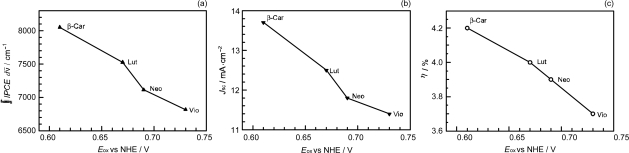
Dependence of **(**a) the integrated *IPCE* (∫*IPCE* d*v̄*), (b) photocurrent (*J*_sc_) and **(c)** conversion efficiency (*η*) of Phe *a*-sensitized solar cells on the one-electron oxidation potential (*E*_ox_) of the Car spacer (reprinted from [18], Copyright (2006), with permission from Elsevier).

Figure 26 exhibits the correlation, ∫*IPCE* d*v̄*, *J_sc_* and *η vs. E_ox_*. It is to be noted that the order of the ∫*IPCE* d*v̄*, *J*_sc_, *η* and *E*_ox_ values and the order in the number of conjugated double bond are reversed between neoxanthin (*n* = 8) and violaxanthin (*n* = 9). As seen in their structures shown in Figure 24, the reversed order originates from the fact that violaxanthin having two electron-withdrawing epoxy groups has higher one-electron oxidation potential than neoxanthin having only one epoxy group. This evidences that the enhancement of the photocurrent and conversion efficiency is determined *not* by the number of conjugated double bonds *but* by the one-electron oxidation potential of the relevant Car spacer.

Finally, we discuss why no singlet-energy transfer was seen even in the present set of plant Cars: The energy diagram in Figure 23 indicates that the 1B_u_^+^ → Q_x_, the 1B_u_^−^ → Q_x_ and the 2A_g_^−^ → Q_y_ singlet energy-transfer pathways should be open for neoxanthin (*n* = 8), and only the 1B_u_^+^ → Q_x_ energy transfer pathway, for violaxanthin (*n* = 9). The results strongly suggest that the 20% Car added, here, as a conjugated spacer may not be enough or the effective distance between the Phe and Car may not be short enough for efficient singlet-energy transfer. Most probably, however, the correct orientation of the transition dipoles is necessary between the Car and Phe *a* moiety. Then, we proceeded to synthesize a Phe–Car adduct so designed.

*Summaries:* A method is found to enhance the photocurrent and conversion efficiency of Phe *a-*sensitized solar cell by the addition of Car as a redox spacer to facilitate the Car to Phe *a*^•+^ electron transfer and to prevent immediate charge recombination in the TiO_2_^−^−Phe *a*^•+^ state. The enhancement increases with the shift of the Car one-electron oxidation potential to the negative side. Subpicosecond pump-probe spectroscopy of Phe *a* and each bacterial Car bound to TiO_2_ nanoparticle in suspension proved that the Car to Phe *a*^•+^ electron transfer actually took place. No clear signs of the Car-to-Phe *a* singlet-energy was seen even in the shortest-chain Cars (*n* = 8).

### 3.2. Pheophorbide–Car adduct: Energy Transfer and Electron Transfer from Car to Phe Moiety

**Figure 27.**
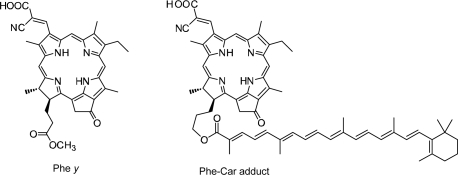
Chemical structures of Phe *y* and Phe–Car adduct (reprinted from [19], Copyright (2007), with permission from Elsevier).

Figure 27 presents the structures of ‘Phe *y*’ sensitizer, *i.e.*, methyl 3^2^-carboxy-3^2^-cyano-pyropherophorbide *a* and ‘Phe–Car adduct’, *i.e.*, 3^2^-carboxy-3^2^-cyano-17^2^- (β-apo-8’-carotenoyl) oxymethyl-17^2^-decarboxy-pyropheophorbide *a*. Phe *y* has a structure similar to Phe *a*, in which the carboxyl group attached to ring A is replaced by the ethenyl-cyano-carboxyl group that was supposed to enhance electron injection. Phe–Car adduct consists of the Phe *y* and *β*-apo-8’-carotenoyl (*n* = 9) moieties.

The *π*-conjugated systems of the two moieties are connected loosely through several single bonds so that their electron clouds can overlap with each other to facilitate efficient electron transfer, and the 1Bu^+^ transition moment of the Car moiety and the Q_x_ transition moment of the Phe moiety can be set parallel to facilitate the 1B_u_^+^ to Q_x_ singlet-energy transfer. When the adduct is bound to the TiO_2_ surface, the intervening bulky Car group may prevent the formation of Phe *y* aggregate and, as a result, suppress the singlet-triplet annihilation reaction.

**Figure 28.**
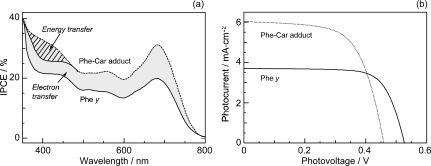
(a) The IPCE profiles and (b) the *I*–*V* curves of solar cells sensitized by Phe *y* and Phe–Car adduct (reprinted from [19], Copyright (2007), with permission from Elsevier).

Figure 28a compares the IPCE profiles of solar cells using the Phe *y* and Phe–Car adduct sensitizers [19]. In the longer-wavelength region (500-800 nm), we see the shift of basically the same IPCE profile from the former to the latter, similar to the cases of bacterial and plant Car spacers (see Figure 19 and Figure 25). In the shorter-wavelength region (370-470 nm), a bump is observed in the IPCE profile of Phe–Car adduct. Definitely, this is ascribable to *singlet-energy transfer* from the Car to Phe *a* moiety. The shift of the IPCE profile in this region is ascribable to *electron transfer* from the Car to the Phe *y* moiety. Figure 28b compares the *I*–*V* curves for the two sensitizers: the Phe *y* sensitizer gives rise to a higher *V*_oc_ value, while the adduct sensitizer, a higher *J*_sc_ value, The former observation presumably reflects the better packing of the Phe *y* sensitizers on the TiO_2_ surface, because the bulky Car moiety in Phe–Car adduct must prevent ordered surface coverage. The latter observation must reflect the larger photo-current due to the electron transfer and energy transfer from the Car to the Phe moiety as mentioned above. Table 1 of Ref. [19] lists the relevant parameters concerning the performance of solar cells using the pair of sensitizers. The introduction of the Car moiety enhances *J*_sc_ by 1.6 times and *η* by 1.3 times. The *E*_ox_ values of Phe–Car adduct reflect those of the Car moiety (0.95 V) and the Phe *y* moiety (1.17 V), which supports the idea of electron transfer from the Car to the Phe moiety.

Again, Figure 23 compares the 1B_u_^+^, 1B_u_^−^ and 2A_g_^−^ energies of Cars and the Q_x_ and Q_y_ energies of Phe *y* (the Q_x_ and Q_y_ absorptions are very similar between Phe *a* and Phe *y*). Energetically, there is a chance of the 1B_u_^+^ → Q_x_ singlet-energy transfer reactions from the Car moiety (*n* = 9) to the Phe *y* moiety. In the energy-transfer reaction, the relative orientation of the transition-dipole moments must be most important, while in the electron-transfer reaction, the overlap of the conjugated systems should be crucial. Both requirements are now satisfied as anticipated from the molecular structure of the Phe–Car adduct (Figure 27).

**Figure 29.**
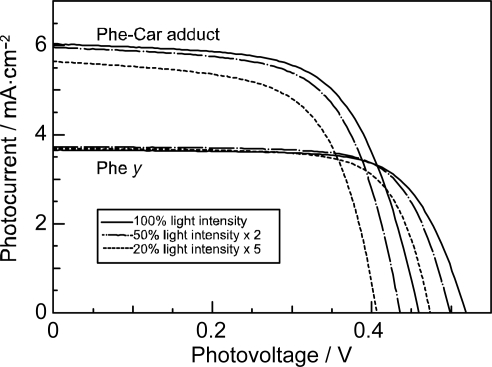
The light-intensity dependence of the *I–V* curves in solar cells using the Phe *y* and Phe–Car adduct sensitizers (reprinted from [19], Copyright (2007), with permission from Elsevier).

Figure 29 compares the light-intensity dependence of the *I*–*V* curves of solar cells using the Phe *y* and Phe–Car adduct sensitizers. In the former, no clear changes in *J*_sc_ is seen even by lowering the light intensity into 1/5, whereas in the latter, systematic decrease in *J*_sc_ is seen as expected. The changes are somewhat comparable to the case of polyenes (see Figure 16): the light-intensity dependence of Phe *y* is similar to that of (MeO)_3_-*ϕ*-6-CA, whereas that of Phe–Car adduct, to that of *ϕ*-6-CA. The results indicate that some aggregation to cause singlet-triplet annihilation is formed in the Phe *y* sensitizer, whereas practically no aggregates are formed in the Phe–Car adduct sensitizer.

Figure 29 pictorially proposes the mechanisms of enhancement in photocurrent and conversion efficiency on going from the Phe *y* to Phe–Car adduct sensitizer, which include (i) electron transfer and (ii) singlet-energy transfer from the Car to the Phe *y* moiety as well as (iii) the suppression of the singlet-triplet annihilation reaction by preventing the aggregate formation by the use of the bulky Car moiety.

*Summary:* Both singlet-energy transfer and electron transfer from the Car to the Phe moiety have been realized in the Phe–Car adduct. The photocurrent (*J*_sc_) was enhanced by 1.6 times, the photovoltage (*V*_oc_) was lowered by 0.9 times and, as a result, the conversion efficiency (*η*) was enhanced by 1.3 times. The π-conjugated chain of the Car moiety prevented the aggregate formation of the Phe moiety so that no sign of singlet-triplet annihilation was seen. Therefore, the Phe–Car adduct is potentially an excellent sensitizer to be used in a more refined way; the addition of short polyene spacers to improve the coverage of the TiO_2_ layer and to enhance the photovoltage (*V*_oc_), for example.

## 4. Bacteriochlorin, Chlorin and Porphyrin Sensitizers

**Figure 31.**
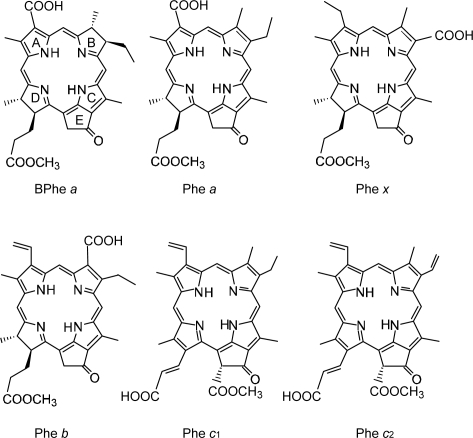
Chemical structure of Phe sensitizers, including BPhe *a* having the bacteriochlorin skeleton, Phe *a*, Phe *x* and Phe *b* having the chlorin skeleton, and Phe *c*_1_ and Phe *c*_2_ having the porphyrin skeleton.

**Figure 32.**
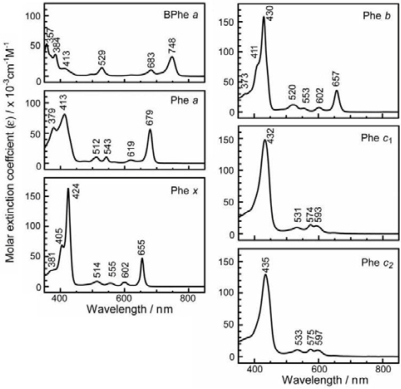
Electronic-absorption spectra of BPhe *a*, Phe *a*, Phe *x*, Phe *b*, Phe *c*_1_ and Phe *c*_2_ in THF solution (reprinted with permission from [20] **^©^** 2008, American Chemical Society).

With the increasing number of conjugated double bonds in the macrocycle one by one, in the order, the bacteriochlorin → chlorin → porphyrin skeleton (see Figure 2), the Soret absorption shifts to the red and the Q_y_ absorption, to the blue, while the relative intensity of absorptions, Soret vs. Q_y_, increases in this order. The structural and spectral changes are shown in Figure 31 and Figure 32, respectively. Thus, the excited-state dynamics, after the photo-excitation, can vary depending on the type of macrocycle. We first examined the photocurrent and conversion efficiency of solar cells using a set of pheophorbide (Phe) sensitizers (with no central metals) having such different types of macrocycle, and found that the performance increased monotonously in the order, Phe *c*_2_ < Phe *c*_1_ < Phe *b* < Phe *x* < Phe *a* ≈ BPhe *a* (*i.e.*, porphyrin < chlorin ≤ bacteriochlorin) with the decreasing one-electron oxidation potential and the increasing Q_y_ absorption.

Next, we examined the photocurrent and conversion efficiency of solar cells using Chl *c*_1_, Chl *c*_2_ and their oxidized derivatives, and found that the introduction of Mg, *i.e.*, Phe *c*_2_ → Chl *c*_2_ (Mg-Phe *c*_2_), for example, substantially enhanced the performance. The results were ascribed to the negative shift of one-electron oxidation potential and, also, to the disappearance of the Q_x_ level that enhances efficient internal conversion from the Soret level.

Finally, we succeeded in enhancing further the performance by co-sensitization, combining the most efficient two sensitizers we have found so far, *i.e.*, Phe *a* and Chl *c*_2_ (Mg-Phe *c*_2_). Also, we have tried to reveal the mechanisms of co-sensitization, suppressing or enhancing the performance of the component sensitizers. The details will be described below.

### 4.1. Pheophorbide Sensitizers Having Bacteriochlorin, Chlorin and Porphyrin Skeletons

**Figure 33.**
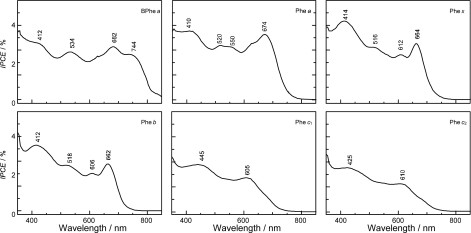
The *IPCE* profiles of solar cells using BPhe *a*, Phe *a*, Phe *x*, Phe *b*, Phe *c*_1_ and Phe *c*_2_ as sensitizers (reprinted with permission from [20] ^©^ 2008, American Chemical Society).

*Dependence of photocurrent and conversion efficiency on one-electron oxidation potential and Q_y_* *absorption.* Figure 31 shows a set of Phe sensitizers, which have different type of skeletons: (a) the bacteriochlorin skeleton in 3-deacetyl-3-carboxy-bacteriopyropoheophobide *a* (BPhe *a*); (b) the chlorin skeleton in methyl 3-carboxy-3-devinyl-pyropheophorbide *a* (Phe *a*), 3-deviny-3-ethyl-8-deethyl-8-carboxy-pyropheo-phorbide *a* (Phe *x*) and methyl 7-deformyl-7-carboxy-pyropheophorbide *b* (Phe *b*); and (c) the porphyrin skeleton in pheophorbides *c_1_* and *c_2_* (Phe *c*_1_ and *c*_2_). We fabricated solar cells using the above set of Phe sensitizers, and compared their photocurrent and conversion efficiencies; we have tried to find *key parameters* that systematically influence the performance by the use of the set of sensitizers with similar structures. Figure 33 shows the IPCE profiles of solar cells using the above set of sensitizers [20], which can be characterized as follows: (i) In BPhe *a*, the IPCE profile is extended to the near-infrared region, a unique property of this sensitizer. (ii) The IPCE profiles in the Q_y_ region are broader in BPhe *a*, Phe *c_1_* and Phe *c_2_* than those in Phe *a*, Phe *x* and Phe *b*. (iii) The IPCE profiles in the Soret region, relative to those in the Q_y_ region, are higher in Phe *x*, Phe *b*, Phe *c_1_* and Phe *c_2_* than in BPhe *a* and Phe *a*. All these characteristics stem from the electronic-absorption spectra of the sensitizers in solution (Figure 32).

**Figure 34.**
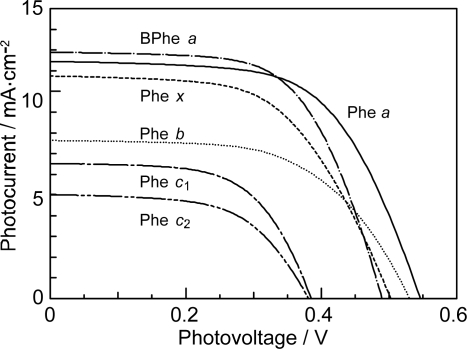
The *I*–*V* curves of solar cells using BPhe *a*, Phe *a*, Phe *x*, Phe *b*, Phe *c*_1_ and Phe *c*_2_ sensitizers (reprinted with permission from [20] ^©^ 2008, American Chemical Society).

Figure 34 shows the *I*–*V* curves of solar cells using the same set of sensitizers. Table 2 of Ref. [20] lists the relevant parameters derived from their IPCE profiles and *I*–*V* curves shown above and below. One-electron oxidation potentials of the sensitizers are also shown in Table 1 of the reference. The photocurrent (*J*_sc_) is in the order, BPhe *a* ≥ Phe *a* > Phe *x* > Phe *b* > Phe *c_1_* > Phe *c_2_*, while the photovoltage (*V*_oc_) is in the order, Phe *a* > Phe *b* > Phe *x* > BPhe *a* >> Phe *c_1_* ≥ Phe *c_2_*. The resultant conversion efficiency (*η*) is in the order, BPhe *a* ≤ Phe *a* > Phe *x* > Phe *b* > Phe *c_1_* > Phe *c_2_*; it is substantially smaller in Phe *c*_1_ and Phe *c*_2_.

**Figure 35.**
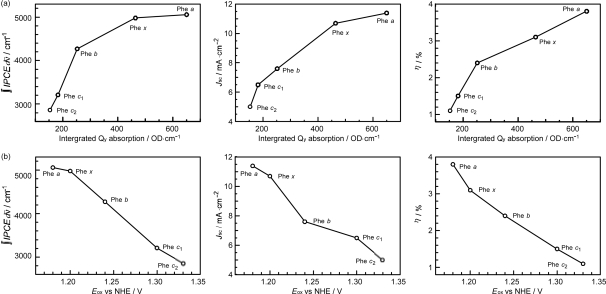
(a) The integrated IPCE (∫ *IPCE* d*v̄*), the photocurrent (*J*_sc_) and the conversion efficiency (*η*) as functions of (a) the integrated Q_y_ absorption and (b) the one-electron oxidation potential (*E*_ox_) for the solar cells using the Phe *a*, Phe *x*, Phe *b*, Phe *c*_1_ and Phe *c*_2_, sensitizers (reprinted with permission from [20] ^©^ 2008, American Chemical Society).

Concerning the five different sensitizers (except for BPhe *a*), both the *J*_sc_ and *η* values are in the order, Phe *a* > Phe *x* > Phe *b* > Phe *c_1_* > Phe *c_2_*. It is important to note that *not* the overall integrated absorption *but* the integrated Q_y_ absorption decreases in the same order. The one-electron oxidation potential (*E*_ox_) of the sensitizer shifts to the positive side in this order, as well. Figure 35a presents the correlation, the integrated incident photon-to-current conversion efficiency (∫*IPCE* d*v̄*), *J*_sc_ and *η* vs. the integrated Q_y_ absorption, whereas Figure 35b presents the correlation, ∫*IPCE* d*v̄*, *J*_sc_ and *η* vs. *E*_ox_. Both correlations exhibit monotonous changes.

Now, we are going to discuss why the photocurrent and, as a result, the conversion efficiency of the solar cells depend on the Q_y_ absorption and the one-electron oxidation potential of the Phe sensitizer.

**Figure 36.**
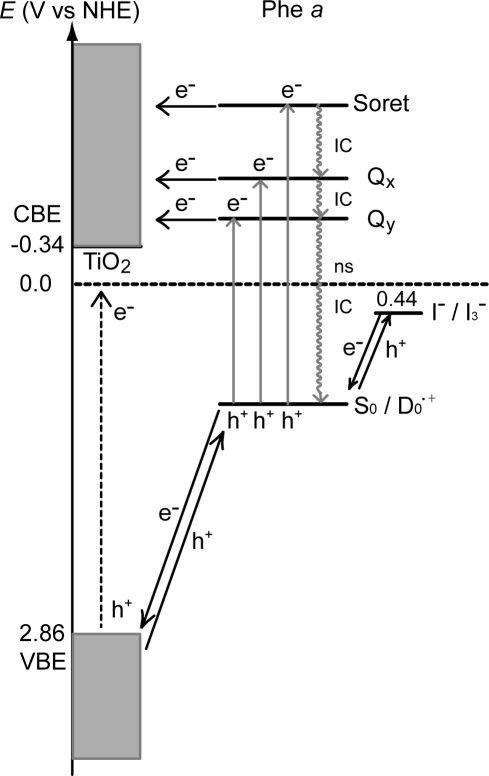
A mechanism of parallel electron transfer, *i.e.*, one, via the singlet-excited states and the other, via the ground redox states.

Figure 36 proposes the parallel flow of electrons in the ground and in the excited states after the excitation of Phe *a* sensitizer: (i) Upon excitation of the dye sensitizer, electron (e^−^) is transferred to an excited state, and, as a result, hole (h^+^) is generated in the ground state. (ii) The electron is injected into TiO_2_, whereas the hole is transferred to the I^−^/I_3_^−^ redox couple. (iii) The latter generates electron flow from the I^−^/I_3_^−^ couple to the radical cation (D_0_^•+^) of the sensitizer. (iv) Upon the UV excitation of TiO_2_, electron transfer from the valence-band edge (VBE) to the conduction-band edge (CBE) can take place. (v) Thus, a parallel flow of electrons, *i.e.*, one, via the excited state and the other, via the ground state can be generated, in principle.

**Figure 37.**
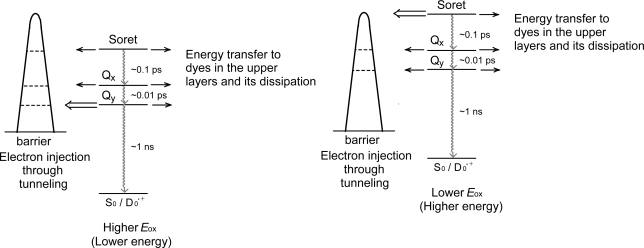
Effects of one-electron oxidation potential on the major electron-injection channels through the tunneling mechanism.

*(A) Dependence on the Q_y_* *absorption*: Figure 37 presents a proposed mechanism of electron injection from the excited states of the Phe sensitizer to the conduction band of TiO_2_ by tunneling through a barrier. The efficiency of electron injection via each excited state, *i.e.*, Soret, Q_x_ or Q_y_, should be determined by competition among (i) electron injection, (ii) internal conversion and (iii) energy transfer to the sensitizer molecules stacked on the upper layers to be dissipated. The key parameter is the rates of internal conversion, which can be assumed to be on the order of (0.1 ps)^−1^, (0.01 ps) ^−1^ and (1 ns) ^−1^ for the Soret, Q_x_ and Q_y_ states, respectively. When the one-electron oxidation potential is *high* (left-hand-side), the barrier is relatively high in energy, and, as a result, the rates of internal conversion and energy transfer via the Soret or the Q_x_ state can be faster than that of electron injection. Then, only the electron injection via the Q_y_ state must play the major role due to its much longer lifetime. Thus, the G → Q_y_ absorption mainly determines the photocurrent,

(1) $$J^{\text{excite}}\;\propto \;Q_{y}\;\text{absorption}$$

*(B) Dependence on the redox potential*: Figure 36 shows that the one-electron oxidation potential determines the relative heights of the Soret, Q_x_ and Q_y_ levels to the barrier for the electron injection. However, since the details of the barrier for electron injection via the excited states are not known at this moment, we will just consider the effect of one-electron oxidation potential on the electron injection *via the ground state* (see Figure 36).

As described in Ref. [20], the following equation can be derived by the use of the Marcus theory:

(2) $$J^{\text{redox}}\;\propto \;\text{exp}\left(-\frac{{\left|\lambda -(2.86-E_{OX})\right|}^{2}}{0.1\lambda }\right).$$

Combining Equations (1) and (2), it turns out that:

(3) $$J_{SC}\;\propto \;J^{\text{excite}}\;+\;J^{\text{redox}}\;=\;A\;+\;B$$

**Figure 38.**
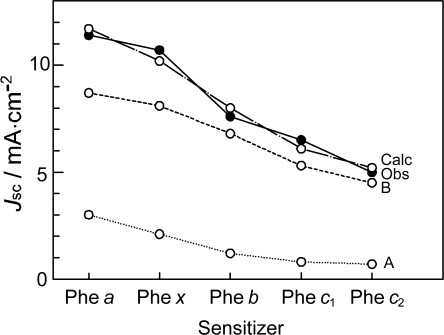
A graphical presentation of the results of fitting to Equation (3) (reprinted with permission from [20] ^©^ 2008, American Chemical Society).

By the use of this equation, we tried to fit the observed values of *J*_sc_ as a function of Q_y_ absorption and *E*_ox_. The numerical fitting results are shown in Table S-1(a) of Supporting Information of Ref. [20]; here, they are presented graphically in Figure 38.

The results apparently indicate that one-electron oxidation potential plays the predominant role. However, this *does not mean* that the electron transfer through the ground redox state is more effective than the electron injection through the Q_y_ state. As mentioned above, the dependence on the one-electron oxidation potential in the electron injection *via the excited states* can be more important than that via the ground state.

**Figure 39.**
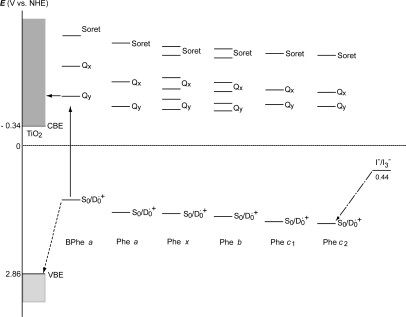
Relative arrangement of the redox ground-state (S_0_/D_0_^•+^) levels and the singlet-excited (Soret, Q_x_ and Q_y_) levels of Phe sensitizers in reference to those of the valence-band-edge (VBE) and the conduction-band-edge (CBE) of TiO_2_ (reprinted with permission from [20] **^©^** 2008, American Chemical Society).

Figure 39 presents the ground-state redox levels (S_0_/D_0_^•+^) and the Q_y_, Q_x_ and Soret excited-state levels of the set of Phe sensitizers in reference to the levels of the valance-band-edge (VBE) and the conduction-band-edge (CBE) of TiO_2_. It can be readily understood that the higher the one-electron oxidation potential (the lower the energy), the less efficient the electron injection via the higher excited states by the electron tunneling mechanism through the barrier (see also Figure 37).

*Summary*: The clear dependence of photocurrent and conversion efficiency on the Q_y_ absorption and the one-electron oxidation potential has been found for the set of Phe sensitizers having the chlorin and porphyrin skeletons, including Phe *a*, Phe *x*, Phe *b*, Phe *c*_1_ and Phe *c*_2_. A fitting result to *J*_sc_ (Figure 38) has been obtained, where *J* ^excite^ reflects the electron injection from the Q_y_ state of Phe sensitizer, and *J* ^redox^ must reflect not only the redox electron transfer in the ground state but also the electron injection from the Q_y_ state through the tunneling mechanism. It is suggested that the idea of electron injection, only through the Q_y_ absorption, originates from the high one-electron oxidation potential (S_0_/D_0_^•+^) of the Phe *c*_2_ sensitizer.

### 4.2. Chl c (Mg-Pheophorbide) Sensitizers Having Porphyrin Skelton

**Figure 40.**
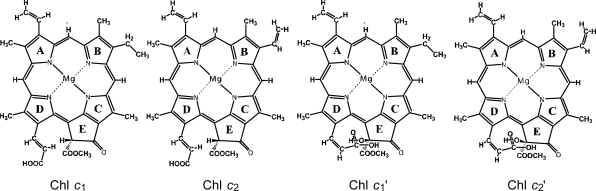
Chemical structures of Chl *c*_1_, Chl *c*_2_, Chl *c*_1_’ and Chl *c*_2_’.

*Solar cells sensitized by Chls c_1_* *and c_2_* *and their oxidized derivatives Chls c_1_’ and c_2_*’. Figure 40 presents the chemical structures of the Chls *c* and Chls *c*’ pairs extracted from a sea weed called ‘*Undaria pinnatifida* (Wakame)’. The structures were determined by mass spectrometry and ^1^H-NMR spectroscopy (including the rotating-frame Overhauser effect spectroscopy (ROESY) measurement to determine the nuclear Overhauser effect (NOE) correlations) [21]: Chl *c*_1_ (Chl *c*_1_’) and Chl *c*_2_ (Chl *c*_2_’) have an ethyl group and a vinyl group, respectively, attached to ring B in different conformations.

Chl *c*_1_ and Chl *c*_2_ (Chl *c*_1_’ and Chl *c*_2_’) have hydrogen (a hydroxyl group) attached to ring E, and also the carboxyl group attached to ring D through the vinyl group in the *trans* (*cis*) conformation with respect to a single bond attached to ring D. Thus, Chl *c*_1_’ and Chl *c*_2_’ can form intramolecular hydrogen bonding between the hydroxyl and carboxyl groups. Importantly, the chemical-shift values of the vinyl H suggest that the electron density is in the order, Chl *c*_2_ > Chl *c*_1_ > Chl *c*_2_’ > Chl *c*_1_’ [21].

**Figure 41.**
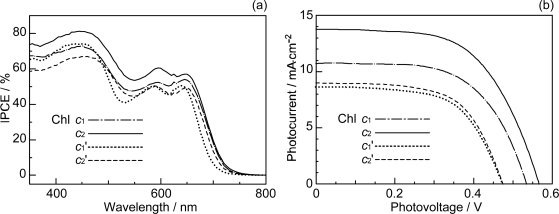
(a) The IPCE profiles and (b) the *I*–*V* curves of solar cells using the Chl *c*_1_, Chl *c*_2_, Chl *c*_1_′ and Chl *c*_2_′ sensitizers (reprinted from [21], Copyright (2007), with permission from Elsevier).

Figures 41a and b show the IPCE profiles and the *I*–*V* curves, respectively, for solar cells using the set of four sensitizers. Table 3 of Ref. [21] lists the relevant parameters derived from the IPCE profiles and the *I–V* curves. The values of ∫*IPCE* d*v̄*, *J*_sc_ and *η* decrease all in the order, Chl *c*_2_ > Chl *c*_1_ > Chl *c*_2_’ ≥ Chl *c*_1_’; the *V*_oc_ value also decreases in the same order. Interestingly, the decreasing order is in agreement with that of the electron density on the vinyl H suggested by the H-chemical-shift values, but not necessarily with that of *the decreasing order* of *E*_ox_, *i.e.*, Chl *c*_1_ > Chl *c*_2_ > Chl *c*_1_’ > Chl *c*_2_’ [21].

**Figure 42.**
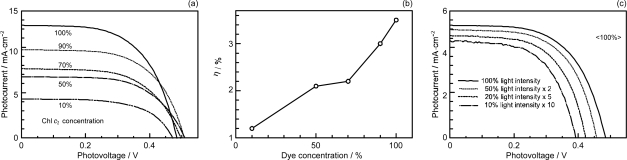
Concentration dependence of the conversion efficiency (*η*), and (c) the light-intensity dependence of the *I*–*V* (a) the *I*–*V* curves, (b) curves in the Chl *c*_2_-sensitized solar cell (reprinted from [21], Copyright (2007), with permission from Elsevier).

Concerning the Chl *c*_2_-sensitizerd solar cell, Figures 42a and b show that the photocurrent (*J*_sc_) and conversion efficiency (*η*) monotonously decrease toward the lower dye concentration, and Figure 42c shows that both the *J*_sc_ and *V*_oc_ values decrease toward the lower light intensity. There is no sign of singlet-triplet annihilation reaction at all due to the aggregate formation in this particular sensitizer.

Chl *c*_2_ has exhibited the highest photocurrent (*J*_sc_ = 13.8 mA·cm^−2^) and conversion efficiency (*η* = 4.6%) among all the sensitizers we have tested. It is rather surprising because Phe *c*_2_ showed one of the lowest photocurrent (*J*_sc_ = 6.0 mA·cm^−2^) and conversion efficiency (*η* = 1.1%), although the absorption spectrum of Chl *c*_2_ (Mg-Phe *c*_2_) to be shown in the next section (Figure 45e, dotted line) is *not* very different from that of Phe *c*_2_ shown in the previous section (Figure 32, bottom). An important difference, however, is the absence and presence of the Q_x_ absorption in the former and the latter, respectively. Most importantly, however, the one-electron oxidation potential of Chl *c*_2_ (1.06 V) is much lower than that of Phe *c*_2_ (1.33 eV).

Here, we will try to explain why the photocurrent and conversion efficiency of the solar cell using the Chl *c*_2_ sensitizer are much higher than those of the solar cell using the Phe *c*_2_ sensitizer in terms of (i) the much lower one-electron oxidation potential and (ii) the absence of the Q_x_ level in the former: Figure 37 shows the effect of lowering the one-electron oxidation potential (on going from the left-had-side to the right-hand-side); then, the excited-state electronic levels shift to the higher energy relative to the barrier. Then, electron injection via the Soret level becomes tremendously enhanced, taking advantage of the highest light absorption efficiency of the Soret absorption. Further, the absence of the Q_x_ level must lengthen the lifetime of the Soret level to enhance the efficiency of electron injection. In addition, the central Mg atom seems to prevent the aggregation of the sensitizer molecules and the resultant singlet-triplet annihilation.

*Summary*. One of the most efficient sensitizer, *i.e.*, Chl *c*_2_, has been found and the mechanism of giving rise to the highest performance has been proposed as mentioned above.

### 4.3. Pheophorbide and Metal-Pheophorbide Sensitizers Having Chlorin and Porphyrin Macrocycles

**Figure 43.**
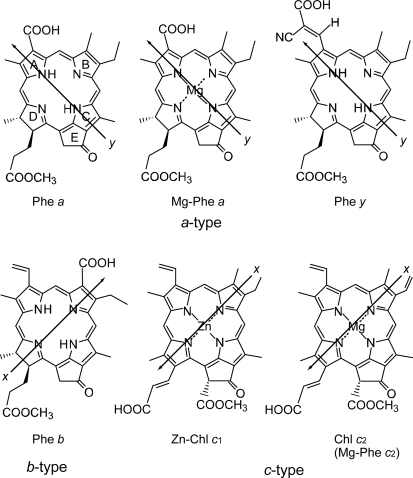
Chemical structures of Phe’s and metal-Phe’s: Phe *a*, Mg-Phe *a*, Phe *y* are classified into the *a*-type sensitizer, Phe *b*, into the *b*-type sensitizer, and Zn-Phe *c*_1_ and Chl *c*_2_ (Mg-Phe *c*_2_), into the *c*-type sensitizer. Each arrow points to the anchoring carboxyl group; directions *x*, *y* are defined in accord with those of the Q_x_ and Q_y_ transitions defined in the chlorin macrocycle of Phe *a*.

*Solar cells sensitized by pheophorbide sensitizers without and with the central metal, Mg or Zn.* Figure 43 presents the structures of sensitizers used in this investigation. The structures can be characterized in two different ways: *(a) The type of macrocycle.* The sensitizers can be classified into *three* different categories (see Figure 43 and Figure 2): (i) Phe *a*, Mg-Phe *a* and Phe *y*, having the chlorin macrocycle like Chl *a*, can be classified into the ‘*a*-type’ sensitizer. (ii) Phe *b* consisting of the chlorin macrocycle, to which a pair of C=O groups is attached in the diagonal positions like Chl *b*, can be classified into the ‘*b*-type’ sensitizer. (iii) Zn-Phe *c*_1_ and Mg-Phe *c*_2_ having the porphyrin macrocycle like Chl *c*, can be classified into the ‘*c*-type’ sensitizer. *(b) The position of the carboxyl group*: The sensitizers can be classified into *two* different groups in terms of the positions of the carboxyl group. (i) The carboxyl group is directly attached to ring A in Phe *a* and Mg-Phe *a*, but through an additional double bond in Phe *y*, (ii) it is attached to ring B in Phe *b*, or it is attached to ring D through a double bond in Zn-Phe *c*_1_ and Chl *c*_2_ (Mg-Phe *c*_2_). In terms of the *x*-axis and the *y*-axis that have been originally defined for the Q_x_ and Q_y_ transitions in Chl *a*, the carboxyl group is on the *y*-axis in Phe *a*, Mg-Phe *a* and Phe *y*, whereas it is on the *x*-axis in Phe *b*, Zn-Phe *c_1_* and Mg-Phe *c_2_* (see the arrows directing to the carboxyl group in the Figure 43).

**Figure 44.**
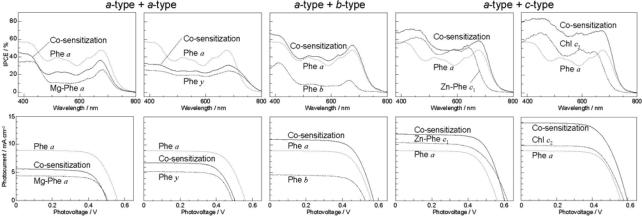
The IPCE profiles (upper panels) and the *I*–*V* curves (lower panels) for three different types of co-sensitization.

Figure 44 exhibits (a) the IPCE profiles and (b) the *I*–*V* curves for the five pairs of sensitizers, which can be classified into *three* different types of co-sensitization, *i.e.*, *a*-type + *a*-type, *a*-type + *b*-type and *a*-type + *c*-type. In the present experiments of co-sensitization, Phe *a* was used as the reference sensitizer. The IPCE profiles and the *I*–*V* curves *pictorially* demonstrate that the co-sensitization of *a*-type + *a*-type gives rise to the suppression, whereas those of *a*-type + *b*-type and *a*-type + *c*-type give rise to the enhancement of photocurrent and conversion efficiency.

Table 5 summarizes the *J*_sc_, *V*_oc_, *FF* and *η* values of the solar cell using the reference Phe *a* sensitizer as well as the pairs of solar cells singly-sensitized by the individual co-sensitizer or co-sensitized with Phe *a*. For co-sensitization, the ratios (*r*) are defined to show the suppression or enhancement of photocurrent and conversion efficiency in reference to the averaged value of the component sensitizers (*^r^J*_sc_ and *^r^η*; see the footnote of the table for their definitions). Concerning the performance of individual co-sensitizer, the chlorin sensitizers can be classified into two groups (here, we abbreviate the relevant units), *i.e.*, Phe *a* with high performance (*J*_sc_ = 9.0, *η* = 3.4), whereas Mg-Phe *a*, Phe *y* and Phe *b* with low performance (*J*_sc_ = 4.4~5.2, *η* = 1.6~1.8). The porphyrin sensitizers give rise to the highest performance, *i.e.*, Zn-Phe *c*_1_ (*J*_sc_ = 10.4, *η* = 4.0) and Chl *c*_2_ (Mg-Phe *c*_2_) (*J*_sc_ = 9.9, *η* = 3.8).

Concerning co-sensitization, the *three* different pairs of sensitizers give rise to suppression or enhancement in reference to the average of performance of the component sensitizers (Table 5): (i) The *a*-type + *a*-type co-sensitization gives rise to suppression of performance; the relative performance values decrease for both sensitizers, *i.e.*, Mg-Phe *a* (*^r^J*_sc_ = 0.83, *^r^η* = 0.76) and Phe *y* (*^r^J*_sc_ = 0.96, *^r^η* = 0.88), the averaged ratios being ~0.8 and ~0.9, respectively. (ii) The *a*-type ^+^ *b*-type co-sensitization with the co-sensitizer, Phe *b*, shows remarkably-high enhancement (*^r^J*_sc_ = 1.60, *^r^η* = 1.65), the averaged ratio being 1.6. (iii) The *a*-type + *c*-type co-sensitization causes large enhancement with the sensitizers, Zn-Phe *c_1_* (*^r^J*_sc_ = 1.23, *^r^η* = 1.35) and Mg-Phe *c_2_* (*^r^J*_sc_ = 1.47, *^r^η* = 1.50), the averaged ratio being ~1.3 and ~1.5, respectively. Importantly, the combination of the chlorin (Phe *a*) and the porphyrin (Mg-Phe *c_2_*) sensitizers, each showing the highest two individual performance (concerning the maximum values ever exhibited), give rise to the highest enhancement of the *J*_sc_ value (9.0 and 9.9 → 14.0 mA·cm^−2^) and the *η* value (3.4 and 3.8 → 5.4%).

**Figure 45.**
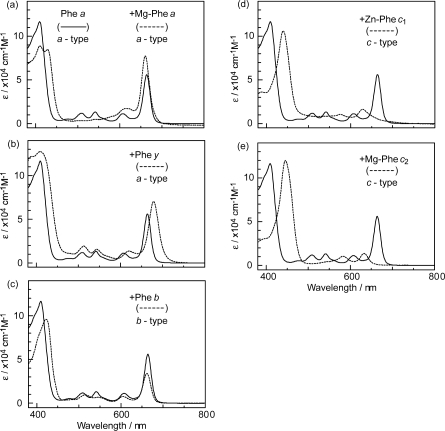
The electronic absorption spectra of the pairs of co-sensitizers in ethanol solution. (a) Phe *c* + Mg-Phe *a*, (b) Phe *a* + Phe *y*, (c) Phe *a* + Phe *b*, (d) Phe *a* + Zn-Phe *c*_1_ and (e) Phe *a* + Mg-Phe *c*_2_.

Figure 45 shows the electronic-absorption spectra of the pairs of sensitizers in ethanol solution, which can be characterized as follows. *Individual sensitizers*: (i) Chlorin sensitizers of both *a*-type (Phe *a* and Phe *y*) and *b*-type (Phe *b*) clearly exhibit the Soret, Q_x_ and Q_y_ absorption peaks, whereas the metal-porphyrin sensitizers of *c*-type (Zn-Phe *a* & Mg-Phe *a*) exhibit *only* the Soret and Q_y_ absorption peaks, the latter of which is split into two. Therefore, completely-different internal conversion processes are expected, *i.e.*, the stepwise Soret → Q_x_ → Q_y_ internal conversion in the *a*-type and *b*-type sensitizer, whereas the direct Soret → Q_y_ internal conversion in the *c*-type sensitizer. (ii) Phe *a* is characterized by a sharp, blue-shifted Soret absorption, whereas the rest of the chlorin sensitizers (Mg-Phe *a*, Phe *y* and Phe *b*) are characterized by a broad, red-shifted Soret absorption. The metal porphyrin sensitizers (Zn-Phe *c*_1_ and Mg-Phe *c*_2_) exhibit a sharp, red-shifted Soret absorption.

*A pair of co-sensitizers*: Depending on the overlapped and split absorption peaks due to the pair of sensitizers, competitive or complementary light absorption is expected to take place. Concerning the overlap of co-sensitizer absorption peaks, (iii) the ‘*a*-type + *a*-type’ co-sensitizer pair exhibits the overlaps of the Soret, Q_x_ and Q_y_ absorptions in a complicated way. (iv) The ‘*a*-type + *b*-type’ pair, *i.e.*, Phe *a* and Phe *b*, exhibits split Soret absorptions, but strongly-overlapped Q_x_ and Q_y_ absorption peaks. (v) The ‘*a*-type + *c*-type’ pair exhibits no overlaps in either the Soret or the Q_y_ absorptions. To evaluate the overlap over the spectral region, we have defined spectral separation (S),

(5) $$S\;=\;\int \left|\varepsilon_{A}(\lambda )-\varepsilon_{B}(\lambda )\right|d\lambda .$$

The values are listed in Table 5. Importantly, it is the smallest in the a-type + b-type pair and the largest in the a-type + c-type pair (see Table 5).

**Figure 46.**
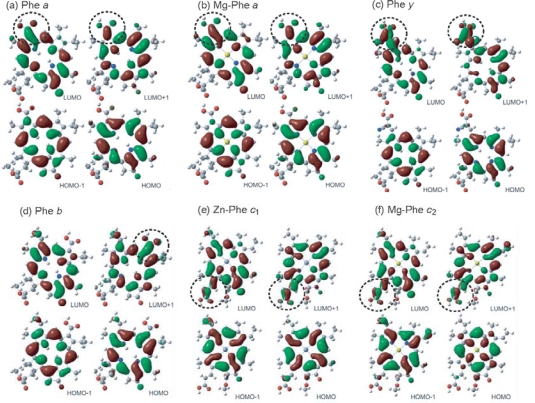
The four orbitals including HOMO–1, HOMO, LUMO and LUMO + 1 obtained by TD-DFT calculations.

We examined the effects due to the type of macrocycles and the position of the carboxyl group on the molecular orbitals by means of the time-dependent density-function-theory (TD-DFT) calculations: Figure 46 shows the calculated four major molecular orbitals, including HOMO–1, HOMO, LUMO and LUMO + 1 (here, HOMO and LUMO stands for the highest-occupied molecular orbital and the lowest-unoccupied molecular orbital, respectively). The shapes of the four molecular orbitals are different depending on the type of macrocycle, chlorin or porphyrin. The LUMO and LUMO+1, that are expected to play the key role in the electron injection into TiO_2_, are found to be extended toward the carboxyl group; in other words, the electron density is shifted toward the carboxyl group to get ready for electron injection (see the regions shown in dotted circles). Also, the electronic transitions are mainly determined by the combination of {HOMO–1, HOMO} → {LUMO, LUMO + 1} transitions and, therefore, all the Soret, Q_x_ and Q_y_ transitions are expected to be strongly influenced by the position of the carboxyl group (or, in other words, by the direction of polarization).

The results of DFT calculations shown in Figure 46 have provided us with a strong support to the ideas that (a) the type of macrocycle, chlorin or porphyrin, and (b) the position of the carboxyl group, on the *y*-axis or the *x*-axis, strongly affect (a) the state energies and the rates of internal conversion and (b) the directions of electron-injection and transition-dipole moment, respectively.

The suppression or enhancement of performance in co-sensitization can be explained in terms of the light absorption (competitive or complementary), the direction of transition-dipole moment (parallel or orthogonal) and the singlet-energy transfer (interactive or independent) between the pair of sensitizers:

- The absorption spectra of the sensitizers (in Figure 45) show that the major light absorption through the Soret bands is highly competitive in the *a*-type + *a*-type pair, complementary rather than competitive in the *a*-type + *b*-type pair, and absolutely complementary in the *a*-type + *c*-type pair. Therefore, the highest enhancement in the *a*-type + *b*-type co-sensitization and the next highest enhancement in the *a*-type + *c*-type co-sensitization can be rationalized in terms of complementary absorption *not* by the Q_x_ and Q_y_ levels *but* by the Soret levels.
- The combination of the *a*-type sensitizer having the carboxyl group in the *y*-direction and the *b*-type or *c*-type sensitizer having the carboxyl group in the *x*-direction should give rise to the highest enhancement of photocurrent and conversion efficiency, because of the minimum interference of the transition dipoles between the pair of co-sensitizers. Polarization and electron-injection along the orthogonal directions must prevent the interference between the intermolecular transition dipole–transition dipole interactions that can trigger intermolecular energy transfer and the resultant dissipation of the singlet energy.
- The different pathways of internal conversion, Soret → Q_x_ → Q_y_ in the *a*-type sensitizer and Soret → Q_y_ in the *b*-type or *c*-type sensitizer may also prevent interaction in the internal-conversion processes because of the different time scales of internal conversion.

*Summary*: Co-sensitization by the use of the best and the second-best sensitizers, *i.e.*, Chl *c*_2_ (Mg-Phe *c*_2_) and Phe *a*, we have achieved the maximum enhancement in photocurrent (*J*_sc_ = 14.0 mA cm^2^) and conversion efficiency (*η* = 5.4%), the enhancement factor being 1.47 and 1.50 times in reference to the averaged value of the performance of the component co-sensitizers. The enhancement is ascribed to the complementary light absorption, the orthogonal transition-dipole moments and the different pathways of internal conversion.

## 5. Conclusions and Future Perspective

### 5.1. Conclusions

- By the use of a set of RA and CA sensitizers (*n* = 5~13), the dependence of photocurrent and conversion efficiency of DSSC on the conjugation-length of the sensitizer was determined to be, in the order, RA5 < CA6 < CA7 > CA8 > CA9 > CA11 > CA13. For comparison, the electron-injection efficiencies for RA5–CA11 bound to TiO_2_ nanoparticles in suspension were determined by means of subpicosecond time-resolved pump-probe spectroscopy. The maximum for CA7 and the decline toward CA11 were explained in terms of excited-state dynamics of the sensitizers. On the other hand, the decline toward RA5 was explained by the increasing efficiency of triplet generation and, as a result, the enhanced singlet-triplet annihilation due to the aggregate formation of the dye sensitizers on the TiO_2_ surface.
- Excited-state dynamics including the formation of a charge-transfer complex, what we call ‘the combined D_0_^•+^ + T_1_ state’, consisting of a charge-separated (TiO_2_^−^–CA (D_0_^•+^) and a neutral (TiO_2_–CA (T_1_)) states, and its subsequent splitting into the D_0_^•+^ plus T_1_ Car species, was identified by subpicosecond and microsecond time-resolved pump-probe spectroscopy, respectively.
- The mechanism of singlet-triplet annihilation to suppress the photocurrent and conversion efficiency was first identified by their dependence on the dye concentration in the CA7-sensitized solar cell. This mechanism was confirmed by the use of sensitizers having the increasing transition-dipole moments and, as a result, the increasing trend of aggregate formation. The least polarizable (the least aggregate-forming) sensitizer gave rise to the decreasing conversion efficiency, whereas the most polarizable (the most aggregate-forming) sensitizer gave rise to the increasing conversion efficiency, both with the decreasing dye concentration and light intensity.
- Sets of bacterial (*n* = 9~13) and plant (*n* = 8~11) Cars were used as redox spacers for the Phe *a*–sensitized solar cell. The idea behind this attempt is to induce electron transfer from Car to Phe *a* radical cation (Phe *a*^•+^) to stabilize the charge-separated TiO_2_^−^– Car^•+^ state to prevent immediate charge recombination of the TiO_2_^−^–Phe *a*^•+^ pair. Rapid electron injection into TiO_2_ to generate Phe *a*^•+^ (20–40 fs) followed by electron transfer from bacterial Cars to Phe *a*^•+^ (200–240 ps) was evidenced by subpicosecond pump-probe spectroscopy of each Phe *a*−bacterial Car pair bound to TiO_2_ nanoparticles in suspension. Among the two set of Cars, *β*-carotene having the lowest one-electron oxidation potential (*E*_ox_ = 0.61 V) exhibited the maximum enhancement of conversion efficiency (*η* = 3.4 → 4.2%). In the above mixture of Car and Phe *a*, no singlet-energy transfer was observed. However, in Phe–Car adduct sensitizer, both singlet-energy transfer and electron transfer from the Car to the Phe moiety were identified in the solar cell. No sign of singlet-triplet annihilation due to aggregate formation was seen in this particular sensitizer.
- In a set of Phe sensitizers having the chlorin and porphyrin macrocycles, photocurrent (*J*_sc_) was found to be the functions of the integrated Q_y_ absorption and one-electron oxidation potential (*E*_ox_). Phe *c*_2_ having the highest one-electron oxidation potential (*E*_ox_ = 1.33 V) exhibited the lowest conversion efficiency (*η* = 1.1%) among the Phe sensitizers. On the other hand, Chl *c*_2_ (Mg-Phe *c*_2_) having low one-electron oxidation potential (*E*_ox_ = 1.06 V) exhibited the highest conversion efficiency (*η* = 4.6%) among all the sensitizers we have tested. The extremely-low conversion efficiency in Phe *c*_2_ was ascribed to the high *E*_ox_ value and electron injection via the Q_y_ level, whereas the high conversion efficiency in Chl *c*_2_ was ascribed to the low *E*_ox_ value and electron injection via the Soret level, which is stabilizer by the absence of the Q_x_ level.
- By co-sensitization using the Phe *a* and Chl *c*_2_ sensitizers of the second-best and the best performance, we have succeeded in enhancing the photocurrent and conversion efficiency to 14.0 mA·cm^−2^ and *η* = 5.4%, respectively. The enhancement was ascribed to the supplementary light absorption, the orthogonal directions of transition-dipoles and the independent internal conversion processes between the pair of sensitizers.

### 5.2. Future Perspective

1. Pump-probe subpicosecond time-resolved spectroscopy of the single Car sensitizer as well as the Chl *a* sensitizer plus Car redox spacer, both bound to TiO_2_ nanoparticles in suspension, has turned out to be very powerful in elucidating the initial electron-injection and electron-transfer mechanisms, respectively. This technique should be applied to determine the mechanisms of excitation, energy-transfer and electron injection in each chlorin or porphyrin sensitizer as well as the pairs of these sensitizers used for co-sensitization.
2. In the case of the well-characterized CA and RA sensitizers, it is time to start pump-probe time-resolved spectroscopy of fabricated DSSCs, in various time regions, to elucidate the real electron flow processes in the cell.
3. To establish the mechanism of singlet-triplet annihilation, which is a key issue to enhance the performance of DSSCs in general, other spectroscopic methods such as time-resolved fluorescence (up-conversion or Kerr-gate) and Raman, in the subpicosecond time region, will be most useful (see Ref. [9], for example).

## 6. Relevant Work by Other Investigators

This mini-review is a condensed summary of our work already published; unpublished results are also included. The authors thought that self-consistent presentation of our own work would make the flow of ideas clear. However, it is fair and appropriate to introduce work by other investigators in the field of DSSCs based on the principles and materials of photosynthesis. Now, we try to introduce the readers most relevant work, which may lead them to a more general perspective and more objective viewpoints on what we have written here. This may also highlight the uniqueness of our work. Here, we follow the order of contents in this review.

*Comparison between DSSC and photosynthesis.* Grätzel [22] presented a summary of DSSC fabricated, which is now called “the Grätzel cell”. In particular, he introduced the microporous structure of sintered TiO_2_ nanoparticles to increase the surface area, mimicing the structure of the thylakoid membrane. The logical construction in developing the first cell module is solid, and this review is an excellent introduction for the beginners to this particular field. Grätzel [23] also published a more detailed description of his DSSC in comparison to plant photosynthesis. We learn that “the Grätzel cell” has been actually build based on “the principle of photosynthesis”.

Cogdell and Lindsay [24] wrote a review addressing whether photosynthesis can provide a ‘biological blueprint’ of novel solar cells. The author has determined, for the first time, the structure of the LH2 antenna complex from a purple bacterium by X-ray crystallography. Therefore, he could describe consistently the structure-function relationship in both the bacterial RC and LH2 antenna complexes. The authors introduced some organic compounds as mimics of the RC and LH2, although their functions when introduced to photovoltaic cells are not clear at this moment.

*A CA9-sensitized solar cell and the excited-state dynamics of RA and CA9 bound to TiO_2_* *nanoparticles in suspension*. Gao *et al.* [25] first fabricated a DSSC using CA9 as a sensitizer and hydroquinone as a reductant. It exhibited IPCE as high as 34% and *V*_oc_ = 0.15 V. Practically, no decay of CA9 on the TiO_2_ surface was observed even after 12 hr.

Pan *et al.* [26] studied the electron injection and charge-recombination mechanisms of CA9 bound to TiO_2_ nanoparticles in suspension by subpicosecond time-resolved pump-probe spectroscopy. They found electron injection from the S_2_ (1B_u_^+^) state with a quantum yield of ~40% but no electron injection from the S_1_ (2A_g_^−^) state. The results are in agreement with ours, except for our value of quantum yield of 60% and our introduction of both the 1B_u_^+^ and 1B_u_^−^ states. They considered two pathways of charge recombination, *i.e.*, one, via the ground state and the other, via the T_1_ state, which may correspond to our proposal of ‘the charge-transfer complex’ labeled state ^3^**3** in Figure 12.

Zhang *et al.* [27] examined RA5 bound to TiO_2_ in suspension by the SVD and global-fitting analysis of subpicosecond time-resolved spectral data. They obtained SADS and time-dependent changes in population similar to ours; they assigned the SADS to the 1B_u_^+^(S_3_), nπ*(S_2_) and 2A_g_^−^ (S_1_) states, whereas we assigned them to the 1B_u_^+^, 2A_g_^−^ and T_1_–D_0_^•+^ states. Concerning the charge-recombination mechanism, they seemed to assume an equilibrium between the radical cation and the T_1_ states as we assumed in the state ‘^3^**3**’ mentioned above. Their conclusions are in general agreement with ours except for the assignment of SADS.

In summary, our unique contribution seems to be the identification of the T_1_–D_0_^•+^ charge-transfer complex (^3^**3**) by the SVD and global-fitting analysis of spectral data in the *μ*s time range.

*Car to radical cation electron transfer in DSSC and photosynthetic systems.* We believed that the addition of Cars to Phe *a*-sensitized solar cells as redox spacers to stabilize the charge-separated state was our own idea, but now we realize that the Car to Chl *a*^•+^ (BChl *a*^•+^) electron transfer is actually one of the principles of photosynthesis: Noguchi *et al.* [28] identified, by FTIR spectroscopy, the generation of *β*-carotene radical cation in photosystem (PS) II membrane at 80 K under the oxidizing condition. Hanley *et al.* [29] studied the oxidation of *β*-carotene in Mn-depleted PS II by means of EPR and electronic-absorption spectroscopy. They proposed possible electron-transfer pathways among the Car, P680, Cyt *b*_559_ and Chl *z*. On the other hand, Polívka *et al.* [30] identified spheroidene radical cation in the LH2 complex from *Rba. sphaeroides.* The detailed mechanisms and function in the photosynthetic systems are still not clear (see [31] for a review), although there is a good chance of the Chl *a*^•+^ and BChl *a*^•+^ generation in the special pair of PS II RC and the B850 aggregate in LH2. In this relation, we should point out that we actually observed the excimer formation of Phe *a*, in our system, prion to the generation of Phe *a*^•+^ (see Ref. [17]).

*Singlet-energy transfer in Car*–*Phe adducts.* Debreczny *et al.* [32] studied singlet-energy transfer in adducts, where two Cars, *i.e.*, fucoxanthin (*n* = 7) and zeaxanthin (*n* = 11), were covalently attached to each of five different pyropheophorbides. In all the five compounds containing fucoxanthin, energy transfer was found to occur from the higher-lying fucoxanthin S_1_ state to the lower-lying pyropheophorbide S_1_ state with the 12–44% efficiency. In contrast, all the five zeaxanthin-containing compounds showed no clear evidence for energy transfer from the zeaxanthin S_1_ state to the pyropheophorbide S_1_ state.

Macpherson *et al.* [33] prepared a model photosynthetic antenna system, consisting of a Car moiety covalently linked to a purpurin to study singlet-energy transfer by means of fluorescence up-conversion spectroscopy. The S_2_ lifetime of 150 ± 3 fs in the isolated Car and that in Car-purpurin dyad of 40 ± 3 fs lead to the energy-transfer efficiency via the S_2_ state, 73 ± 6%. On the other hand, the S_1_ lifetime of Car (7.8 ps) was not changed at all even after the formation of the dyad. Taken together, the S_2_ state of the Car moiety is concluded as the sole donor state in the singlet-energy transfer.

Polívka *et al.* [34] examined the Car to pyropheophorbide singlet energy transfer for dyads containing carboxyl Cars, peridinin (dyad 1) and fucoxanthin (dyad 2). Energy transfer occurred in 31–44 ps for dyad 1, whereas in 195–280 ps in dyad 2. Energy-transfer efficiency varied with solvent polarity: 80% in benzene, 69% in tetrahydrofuran and 22% in acetonitrile for dyad 1, whereas 27% in benzene, 18% in tetrahydrofuran and 13% in acetonitrile for dyad 2.

Those model antennas can be used as a guide for designing the dyad sensitizers in the future, after adding the carboxyl group for the binding and electron injection to TiO_2_.

*Chlorin and porphyrin sensitizers*. As a pioneering work in the usage of Chl derivatives and related porphyrins, Kay and Grätzel [35] found that compounds containing copper as the central metal gave rise to the highest IPCEs. Cu mesoporphyrin IX exhibited an IPCE value as high as 83% at the Soret absorption, *i.e.*, a unit quantum yield of charge separation when the loss of light energy by reflection and scattering was taken into account. On the other hand, Cu chlorophyllin gave rise to performance with a *J*_sc_ value of 9.4 mA cm^−2^, a *V*_oc_ value of 0.52 V, and the resultant *η* value of 2.6%. It was found that the conjugation of the carbonyl group with the π electron system of the chromophore was not absolutely necessary, and that cholanic acids as co-adsorbates were useful to improve the photocurrent and photovoltage of solar cells using those sensitizers.

Nazeeruddin *et al.* [36] show that Zn porphyrins exhibit much better performance than Cu porphyrins. Tetraporphyrinato Zn(II) ethenyl benzoic acid showed the best performance as the sensitizer, *i.e.*, *J*_sc_ = 9.7 mA cm^−2^, *V*_oc_ = 0.66 V and *η* = 4.8%. Campbell *et al.* [37] compared the performance of a wide variety of porphyrins to reveal structural dependence. They found this compound was the best as a sensitizer.

Wang *et al.* [38] examined a series of metalloporphyrins and found that the **Zn**-**3**-sensitized solar cell demonstrated high performance with *J*_sc_ = 13.0 ± 0.5 mA cm^−2^, *V*_oc_ = 0.61 ± 0.5 mV, and *η* = 5.6%. Most recently, Campbell *et al.* [39] reported a porphyrin sensitized solar cell of extremely-high performance, *i.e.*, *J*_sc_ = 14.0 ± 0.20 mA cm^−2^, *V*_oc_ = 0.68 ± 0.03 V, and *η* = 7.1%, the most efficient porphyrin-sensitized solar cell reported to date.

The performance of our DSSCs may have been underestimated due to our fabricating technique. The conversion efficiency of the Phe *a*-sensitized cell exhibited *η* = 3.4% when we fabricated, but a DSSC fabricated by Dr. Nazeeruddin, by the use of the same sensitizer, showed the value as high as *η* = 5.1% (personal communication). Assuming ‘the technical factor’ of 5.1/3.4 = 1.5, the conversion efficiencies for solar cells sensitized by Chl *c*_2_ and co-sensitized by Phe *a* + Chl *c*_2_ turn out to be 3.8 × 1.5 = 5.7 and 5.4 × 1.5 = 8.1%, respectively. Obviously, we need to improve our technique of solar-cell fabrication to *correctly determine* the conversion efficiency for each sensitizer.

## Figures and Tables

**Figure 15.**
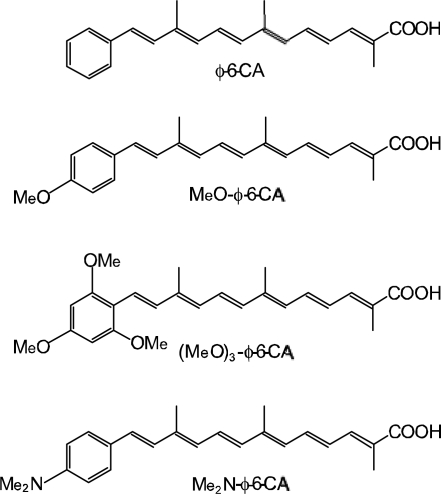
Chemical structures of a set of four polyene sensitizers with the increasing polarizability and, as a result, the increasing tendency of aggregate formation (reprinted from [15], Copyright (2006), with permission from Elsevier).

**Figure 30.**
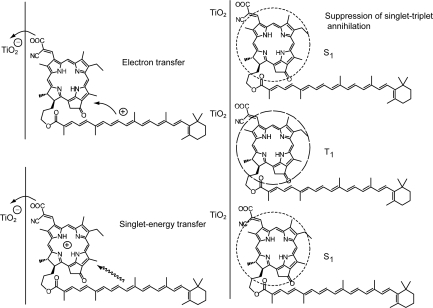
Mechanisms of enhancement of photocurrent and conversion efficiency (as indicated) in the solar cell using the Phe–Car adduct sensitizer (reprinted from [19], Copyright (2007), with permission from Elsevier).

**Table 1.** Electron-injection efficiencies (in %) through the 1B_u_^+^ and the 2A_g_^−^ channels and a sum of them calculated by the use of time constants shown in Figure 9 (reprinted with permission from [11] **^©^** 2005, American Chemical Society).

	**RA5**	**CA6**	**CA7**	**CA8**	**CA9**	**CA11**
**1B_u_^+^ channel**	0.04	0.31	0.46	0.31	0.60	0.29
**2A_g_^−^ channel**	0.88	0.61	0.52	0.63	—	—
**Sum**	0.92	0.92	0.98	0.94	0.60	0.29

**Table 2.** One-electron oxidation potentials in dichloromethane (in V) (reprinted with permission from [11] **^©^** 2005, American Chemical Society).

	**RA5**	**CA6**	**CA7**	**CA8**	**CA9**	**CA11**
***E*_ox_ (*vs*. Ag/AgCl)**	1.08	0.97	0.87	0.80	0.77	0.71

**Table 3.** The time constants of transformation from the D_0_^•+^ – T_1_ complex to the D_0_^•+^ and T_1_ states (*k*_d_^−1^ and *k*_t_^−1^) and the D_0_^•+^ and T_1_ lifetimes (*k*_d0_^−1^ and *k*_t0_^−1^). The partition efficiencies from the D_0_^•+^ – T_1_ complex to the D_0_^•+^ and T_1_ states (*ϕ*_D_ and *ϕ*_T_) are also listed (reprinted with permission from [11] **^©^** 2005, American Chemical Society).

	**RA5-TiO_2_**	**CA6-TiO_2_**	**CA7-TiO_2_**	**CA8-TiO_2_**
***k*_d_^−1^(μs)**	34	22	9.4	5.9
***k*_t_^−1^ (μs)**	3.1	2.7	2.1	2.0
***k*_t0_^−1^ (μs)**	22	18	12	9.0
***k*_d0_^−1^ (μs)**	~50	~150	~150	~150
*ϕ*_D_**(%)**	8	11	18	25
*ϕ*_T_**(%)**	92	89	82	75

**Table 4.** Time constants (in ps) determined by the SVD and global-fitting analyses (reprinted from [17], Copyright (2005), with permission from Elsevier).

**Time constant**	**no Car**	+**Neu**	+**Sph**	+**Lyc**	+**Ahr**	+**Spx**
**Phe*a*^• +^rise**	0.03	0.04	0.03	0.03	0.02	0.02
**Phe*a*^• +^ decay**	338	203	210	220	235	241

**Table 5.** The open-circuit photovoltage (*V*_oc_), fill factor (*FF*), short-circuit photo-current density (*J*_sc_), conversion efficiency (*η*) of the solar cells singly-sensitized and co-sensitized, and the spectral separation (S) and one-electron oxidation potential (*E*_ox_) of the sensitizers.

**Principal and Co-sensitizers**	***V*_oc_/V**	**FF**	***J*_sc_(Av)**^*r*^***J*_*sc*_/mA·cm^−2^**			***η*(Av)**^*r*^***η*/%**			$\frac{{}^{r}J_{sc}+{}^{r}\eta }{2}\;\text{S}$**S**		***E*_ox_/V_vs_**
Phe *a* (*a*-*type*) *a-type*	0.56	0.68	9.0			3.4					1.16
Mg-Phe *a*	0.51	0.70	4.4			1.6					0.79
co-sensitization	0.50	0.69	5.6	(6.7)	0.83	1.9	(2.5)	0.79	0.8	41	
Phe *y*	0.49	0.70	5.2			1.8					1.19
co-sensitization *b-type*	0.50	0.68	6.8	(7.1)	0.36	2.3	(2.6)	0.92	0.9	62	
Phe *b*	0.53	0.70	4.6			1.7					1.24
co-sensitization *c-type*	0.57	0.68	10.9	(6.8)	1.60	4.3	(2.6)	1.65	1.6	39	
Zn-Phe *c*_1_	0.62	0.63	10.4			4.0					1.16
co-sensitization	0.60	0.69	11.9	(9.7)	1.23	5.0	(3.7)	1.35	1.3	80	
Mg-Phe *c*_2_ (Chl *c*_2_)	0.58	0.66	9.9			3.8					1.06
co-sensitization	0.60	0.64	14.0	(9.5)	1.47	5.4	(3.6)	1.50	1.5	95	

The ratio of *J_sc_*: ${}^{r}J_{sc}\;=\;\frac{J_{sc}(A-B)}{\{J_{sc}(A)+J_{sc}(B)\}/2}$; The ratio of *η*: ${}^{r}\eta \;=\;\frac{\eta (A-B)}{\{\eta (A)+\eta (B)\}/2}$

## References

[1] Furuichi K Sashima T Koyama Y The first detection of the 3A_g_^−^ state in carotenoids using resonance-Raman excitation profiles Chem. Phys. Lett 2002 356 547 555

[2] Rondonuwu FS Taguchi T Fujii R Yokoyama K Koyama Y Watanabe Y The energies and kinetics of triplet carotenoids in the LH2 antenna complexes as determined by phosphorescence spectroscopy Chem. Phys. Lett 2004 384 364 371

[3] Zhang J-P Fujii R Koyama Y Rondonuwu FS Watanabe Y Mortensen A Skibsted LH The 1B_u_–type singlet state of *β*-carotene as a precursor of the radical cation found in chloroform solution by sub-picosecond time-resolved absorption spectroscopy Chem. Phys. Lett 2001 348 235 241

[4] Hoff AJ Amesz J Visible absorption spectroscopy of chlorophylls Chlorophylls Scheer H CRC Press, Inc Boca Raton, FL, USA 1991 723 738

[5] Gouterman M Spectra of porphyrins J. Mol. Spectrosc 1961 6 138 163

[6] Gouterman M Wagnière GH Snyder LC Spectra of porphyrins: Part II. Four orbital model J. Mol. Spectrosc 1963 11 108 127

[7] Hanson LK Molecular orbital theory of monomer pigments Chlorophylls Scheer H CRC Press, Inc Boca Raton, FL, USA 1991 993 1014

[8] Koyama Y Rondonuwu FS Fujii R Watanabe Y Light-harvesting function of carotenoids in photosynthesis: The roles of the newly found 1^1^B_u_^−^ state Biopolymer 2004 74 2 18 10.1002/bip.20034 15137086

[9] Koyama Y Kakitani Y Watanabe Y Photophysical properties and light-harvesting and photoprotective functions of carotenoids in bacterial photosynthesis: Structural selections Primary Processes of Photosynthesis, Part 1: Principles and Apparatus (Comprehensive Series in Photochemical and Photobiology vol 8) Renger G RSC Publishing Cambridge, UK 2008 151 201

[10] Wang X-F Fujii R Ito S Koyama Y Yamano Y Ito M Kitamura T Yanagida S Dye-sensitized solar cells using retinoic acid and carotenoic acids: Dependence of performance on the conjugation length and the dye concentration Chem. Phys. Lett 2005 416 1 6

[11] Xiang J-F Rondonuwu FS Kakitani Y Fujii R Watanabe Y Koyama Y Mechanisms of electron injection from retinoic acid and carotenoic acids to TiO_2_ nanoparticles and charge recombination via the T_1_ state as determined by subpicosecond to microsecond time-resolved absorption spectroscopy: Dependence on the conjugation length J. Phys. Chem. B 2005 109 17066 17077 16853176 10.1021/jp051480p

[12] Fujii R Inaba T Watanabe Y Koyama Y Zhang J-P Two different pathways of internal conversion in carotenoids depending on the length of the conjugated chain Chem. Phys. Lett 2003 369 165 172

[13] Tavan P Schulten K Electronic excitations in finite and infinite polyenes Phys. Rev. B 1987 36 4337 4358 10.1103/physrevb.36.4337 9943414

[14] Dung D Ramsden J Graetzel M Dynamics of interfacial electron-transfer processes in colloidal semiconductor systems J. Am. Chem. Soc 1982 104 2977 2985

[15] Wang X-F Koyama Y Nagae H Yamano Y Ito M Wada Y Photocurrents of solar cells sensitized by aggregate-forming polyenes: Enhancement due to suppression of singlet-triplet annihilation by lowering of dye concentration or light intensity Chem. Phys. Lett 2006 420 309 315

[16] Wang X-F Xiang J-F Wang P Koyama Y Yanagida S Wada Y Hamada K Sasaki S Tamiaki H Dye-sensitized solar cells using a chlorophyll *a* derivative as the sensitizer and carotenoids having different conjugation lengths as redox spacers Chem. Phys. Lett 2005 408 409 414

[17] Wang X-F Kakitani Y Xiang J-F Koyama Y Rondonuwu FS Nagae H Sasaki S Tamiaki H Generation of carotenoid radical cation in the vicinity of a chlorophyll derivative bound to titanium oxide, upon excitation of the chlorophyll derivative to the Q_y_ state, as identified by time-resolved absorption spectroscopy Chem. Phys. Lett 2005 416 229 233

[18] Wang X-F Matsuda A Koyama Y Nagae H Sasaki S Tamiaki H Wada Y Effects of plant carotenoid spacers on the performance of a dye-sensitized solar cell using a chlorophyll derivative: Enhancement of photocurrent determined by one electron-oxidation potential of each carotenoid Chem. Phys. Lett 2006 423 470 475

[19] Wang X-F Koyama Y Wada Y Sasaki S Tamiaki H A dye-sensitized solar cell using pheophytin-carotenoid adduct: Enhancement of photocurrent by electron and singlet-energy transfer and by suppression of singlet-triplet annihilation due to the presence of the carotenoid moiety Chem. Phys. Lett 2007 439 115 120

[20] Wang X-F Koyama Y Nagae H Wada Y Sasaki S Tamiaki H Dependence of photocurrent and conversion efficiency of titania-based solar cell on the Q_y_ absorption and one electron-oxidation potential of pheophorbide sensitizer J. Phys. Chem. C 2008 112 4418 4426

[21] Wang X-F Zhan C-H Maoka T Wada Y Koyama T Fabrication of dye-sensitized solar cells using chlorophylls *c*_1_ and *c*_2_ and their oxidized forms *c*_1_’ and *c*_2_’ from *Undaria pinnatifida* (Wakame) Chem. Phys. Lett 2007 447 79 85

[22] Grätzel M The artificial leaf, molecular photovoltaics achieve efficient generation of electricity from sunlight Comment. Inorganic. Chem 1991 12 93 111

[23] Grätzel M The artificial leaf, bio-mimetic photocatalysis CATTECH 1999 3 4 17

[24] Cogdell RJ Lindsay JG Can photosynthesis provide a ‘biological blueprint’ for the design of novel solar cells? TIBTECH 1998 16 521 527

[25] Gao FG Bard AJ Kispert LD Photocurrent generated on a carotenoid-sensitized TiO_2_ nanocrystalline mesoporuos electrode J. Photochem. Photobiol. A: Chem 2000 130 49 56

[26] Pan J Benkō G Xu Y Pascher T Sun L Sundström V Polívka T Photoinduced electron transfer between a carotenoid and TiO_2_ nanoparticle J. Am. Chem. Soc 2002 124 13949 13957 12431127 10.1021/ja0279186

[27] Zhang L Yang J Wang L Yang G-Z Weng Y-X Direct observation of interfacial charge recombination to the excited-triplet state in all-*trans*-retinoic acid sensitized TiO_2_ nanoparticles by femtosecond time-resolved difference absorption spectroscopy J. Phys. Chem. B 2003 107 13688 13697

[28] Noguchi T Mitsuka T Inoue Y Fourier transform infrared spectrum of the radical cation of *β*-carotene photoinduced in photosystem II FEBS Lett 1994 356 179 182 7805833 10.1016/0014-5793(94)01263-6

[29] Hanley J Deligiannakís Y Pascal A Faller P Rutherford AW Carotenoid oxidation in photosystem II Biochemistry 1999 38 8189 8195 10387064 10.1021/bi990633u

[30] Polívka T Zigmantas D Herek JL He Z Pascher T Pullerits T Cogdell RJ Frank HA Sundström V The carotenoid S_1_ state in LH2 complexes from purple bacteria *Rhodobacter sphaeroides* and *Rhodopseudomonas acidophila*: S_1_ energies, dynamics, and carotenoid radical formation J. Phys. Chem. B 2002 106 11016 11025

[31] Frank HA Brudvig GW Redox functions of carotenoids in photosynthesis Biochemistry 2004 43 8607 8615 15236568 10.1021/bi0492096

[32] Debreczeny MP Wasielewski MR Shinoda S Osuka A Singlet-singlet energy transfer mechanisms in covalently-linked fucoxanthin- and zeaxanthin-pyropheophorbide molecules J. Am. Chem. Soc 1997 199 6407 6414

[33] Macpherson AN Liddell PA Kuciauskas D Tatman D Gillbro T Gust D Moore TA Moore AL Ultrafast energy transfer from a carotenoid to a chlorin in a simple artificial photosynthetic antenna J. Phys. Chem. B 2002 106 9424 9433

[34] Polívka T Pellnor M Melo E Pascher T Sundström V Osuka A Naqvi KR Polarity-tuned energy transfer efficiency in artificial light-harvesting antenna containing carbonyl carotenoids peridinin and fucoxanthin J. Phys. Chem. B 2007 111 467 476

[35] Kay A Grätzel M Artificial photosynthesis. 1. Photosensitization of TiO_2_ solar cells with chlorophyll derivatives and related natural porphyrins J. Phys. Chem 1993 97 6272 6277

[36] Nazeeruddin MdK Humphry-Baker R Officer DL Campbell WM Burrell AK Grätzel M Application of metalloporphyrins in nanocrystalline dye-sensitized solar cells for conversion of sunlight into electricity Langmuir 2004 20 6514 6517 15248744 10.1021/la0496082

[37] Campbell WM Burrell AK Officer DL Jolley KW Porphyrins as light harvesters in the dye-sensitised TiO_2_ solar cell Coord. Chem. Rev 2004 248 1363 1379

[38] Wang Q Campbell WM Bonfantani EE Jolley KW Officer DL Walsh PJ Gordon K Humphry-Baker R Nazeeruddin MK Grätzel M Efficient light harvesting by using green Zn-porphyrin-sensitized nanocrystalline TiO_2_ films J. Phys. Chem. B 2005 109 15397 15409 16852953 10.1021/jp052877w

[39] Campbell WM Jolley KW Wagner P Wagner K Walsh PJ Gordon KC Schmidt-Mende L Nazeeruddin MK Wang Q Grätzel M Officer DL Highly efficient porphyrin sensitizers for dye-sensitized solar cells J. Phys. Chem. C 2007 111 11760 11762

